# Introducing a green nanocatalytic process toward the synthesis of benzo[*a*]pyrano-[2,3-*c*]phenazines utilizing copper oxide quantum dot-modified core–shell magnetic mesoporous silica nanoparticles as high throughput and reusable nanocatalysts†

**DOI:** 10.1039/d2ra03887k

**Published:** 2022-09-05

**Authors:** Mohaddeseh Dehnavian, Abdulhamid Dehghani, Leila Moradi

**Affiliations:** Department of Organic Chemistry, Faculty of Chemistry, University of Kashan Kashan Iran P.O. Box 8731753153 l_moradi@kashanu.ac.ir +983155912336

## Abstract

In this contribution, a green, simple, efficient, and straightforward nanocatalytic process was developed for the synthesis of benzo[*a*]pyrano[2,3-*c*]phenazine derivatives under mild thermal conditions. In this regard, the copper oxide quantum dot-modified magnetic silica mesoporous nanoparticles (M-MSNs/CuO(QDs)) were synthesized by surface modification of M-MSNs with CuO QDs to prepare a highly powerful magnetic core–shell nanocatalyst. The prepared nanocatalyst was then characterized for its functionality, size, morphology, elemental composition, surface area, crystallinity, and magnetic properties. Afterwards, it was applied for the synthesis of benzo[*a*]pyrano[2,3-*c*]phenazine derivatives under green reaction conditions. The factors affecting the reaction yield were optimized by the one-factor-at-a-time optimization method. Under obtained optimal conditions, the developed method showed a reaction yield range as high as 86–95% for different derivatives. The reusability studies were performed for indexing the cycling stability of the prepared magnetic nanocatalyst. The results exhibited that the catalytic efficiency of the nanocatalyst was saved for at least 5 operational times, showing high cycling stability of M-MSNs/CuO(QDs). Finally, the catalytic performances of the nanocatalyst was compared with the reported ones, revealing that the M-MSNs/CuO(QDs) presents very better performances toward the synthesis of benzo[*a*]pyrano[2,3-*c*]phenazine derivatives than the reported ones.

## Abstract 2

In this contribution, a green, simple, efficient, and straightforward nanocatalytic process was developed for the synthesis of benzo[*a*]pyrano[2,3-*c*]phenazine derivatives under mild thermal conditions.

## Graphical abstract

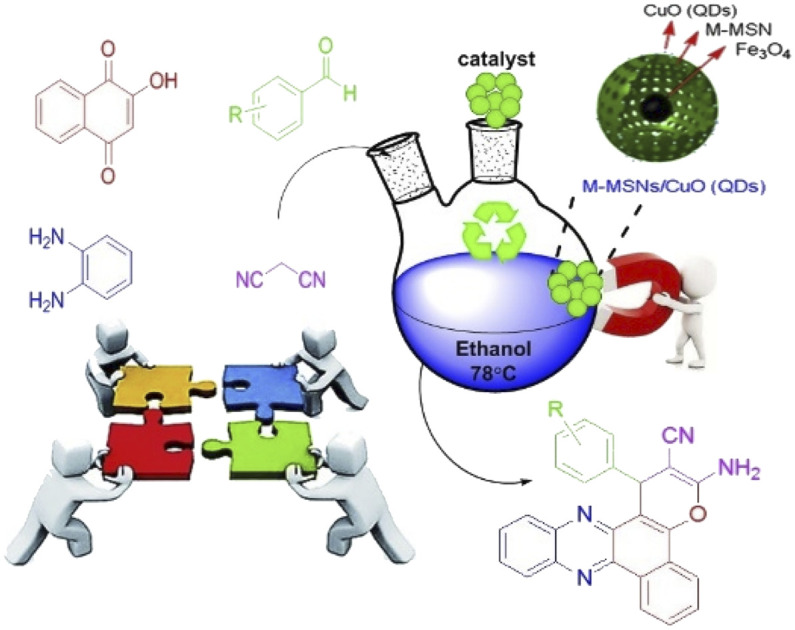

## Introduction

Fluorescent semiconductor nanostructures with quantum properties are known as quantum dots (QDs).^1^ Basically, QDs are a type of nanoarchitecture that is produced from semiconductor atoms of group II and VI elements such as CdTe and CdSe or atoms of group III and V elements, for instance InAs and InP.^2^ The QDs show several unique physicochemical characteristics which can be assigned to the generation of multiple excitons and quantum confinement effects.^1–3^ These nanostructures have a diameter from subnanometers to a few nanometers and revealed size-dependent optical (*i.e.*, absorption and emission) properties.^3^ The size-dependent electronic properties of QDs permit researchers to control their optical characteristics by tuning their size. The tunable electronic properties of QDs lead to their wide applications in several fields for example, in sensing and detection,^4,5^ photovoltaics, bioimaging,^6^ and drug delivery,^7^ as well as catalysis.^8,9^ QDs are considered ideal catalysts and probes due to their size uniformity, the tunability of their optical features, facile surface modification, easy production, excellent catalytic features, and high stability.^10^ Among these QDs, the well-known covalent semiconductor cupric oxide quantum dots (CuO QDs) have been widely used for several applications, for instance, catalysis and photo-catalysis, solar cell development, and sensor design due to their availability, cost-efficiency, narrow bandgap, and magnetic and optical features.^7^

As it is well-known, the high surface area of the catalysts is one of the most important characteristics in their application toward catalytic reactions with a combined adsorption-catalysis mechanism. In other words, catalysts supported by porous materials are more attractive than non-supported catalysts due to their excellent advantages, for instance, large pore volume, large specific surface area, as well as uniform and tunable pore size.^11–13^ In this regard, the silica mesoporous nanoparticles with high surface area and excellent porosity are good choices for enhancing the photocatalytic properties of the catalysts compared to their non-supported analogs.^13^ Moreover, the silica can protect the QDs against environmental forces such as pH variations, and high salt concentrations, as reported.^10^ Besides, the ease of handling of a catalyst is a great advantage for its application in commercial or even industrial processes. In this regard, the magnetic nanoparticles and their composites exhibited a unique attraction for use as reusable and easy to handle catalysts.^14^

Since 1850, with the initial report on the Strecker synthesis of α-amino cyanides, multi-component reactions (MCRs), or in other words, the multi-component assembly process (MCAP), have been introduced as a powerful strategy for synthesizing biologically active substances as well as highly effective bonding tools in organic and pharmaceutical chemistry.^15–17^ One of the reasons for the use of these reactions is the interest of chemists in the preparation and synthesis of small bioactive molecules and pharmaceutical compounds. In addition, the structural complexity of products in these reactions offers unique potential in pharmaceutical research for newer bioactive compounds.^18,19^ Nowadays, the concepts of multi-component processes as masterpieces of synthetic efficiency and reaction design in the synthesis of complex and highly diverse structures have led to the design and implementation of MCRs in universities and industry. Unique features of multicomponent reactions include cheapness, availability of materials, structural variability with an increasing number of reactants, high efficiency and short reaction time, development of drugs through domino reactions and parallel synthesis.^20–22^ It should be noted that in recent years, multi-component reactions have been used to synthesize a wide range of analogues belonging to various classes of heterocyclic compounds.^23^

In 1859, after the discovery of safranin by Greville Williams and its commercial use, it became clear that phenosafranine was a phenazine-containing system. Phenazines are heterocyclic compounds containing nitrogen, and natural phenazine products can be considered as secondary metabolites derived from a primary metabolite that form the core of many natural and synthetic organic matter, in other words, phenazines are colored aromatic secondary metabolites containing nitrogen, which are produced by different species of bacteria. Natural phenazine products are isolated from the secondary metabolites Pseudomonas, Streptomyces or marine habitats. Phenazines are the major constituents of many dyes, such as safranins, toluylene red, and indolines.^24–27^ These compounds have biological properties such as antibiotic, anti-tumor, anti-malarial and anti-parasitic properties.^26,28–30^ The unique applications of phenazines are included to: charge transfer due to electron-rich chromophores, increasing the bacterial lifespan, using as electron shuttles to replace terminal receivers, changes in cellular redox states and function as cellular signals and regulate gene expression patterns.^26,31^

**Scheme 1:**
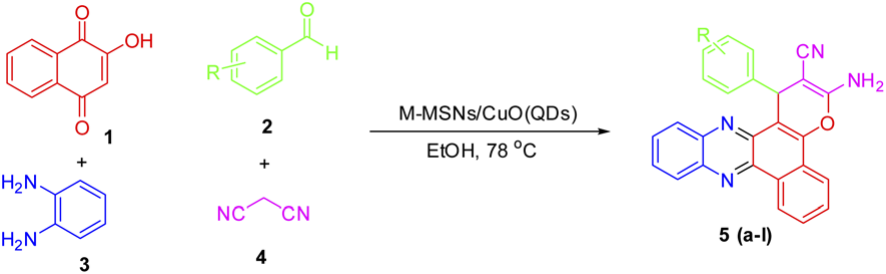
Preparation of benzo[*a*]pyrano-[2,3-*c*]phenazines using M-MSNs/CuO(QDs).

Benzo[*a*]phenazines that have the structure of naphthoquinone and phenazine in their structure are introduced as anti-tumor agents and dual topoisomerase inhibitors.^32^ In recent years, several methods have been reported for the synthesis of benzo[*a*]pyrano[2,3-*c*]phenazines using various catalysts such as pyridine, DABCO, ionic liquids, theophylline, acetic acid, nano copper oxide, cyclodextrin, and triethylamine.^24,33–38^ Reported catalysts have disadvantages such as corrosiveness, high heat capacity and boiling point, and non-recyclability therefore, the importance of phenazines and naphthoquinones will lead to finding synthetic processes based on green chemistry protocols for the synthesis of these organic compounds. Hence, in this paper, the use of CuO QDs in combination with magnetic silica mesoporous nanoparticles is considered to prepare an efficient, high stable, reusable, and easy to handle core–shell nanocatalyst. On the other hand, we evaluate its catalytic properties for the development of a green, simple, efficient nanocatalytic process for the synthesis of benzo[*a*]pyrano-[2,3-*c*]phenazine derivatives under mild thermal conditions (Scheme 1).

## Experimental section

### Chemicals and instrumentation

Chemicals were purchased from Merck Chemical Company in high purity and were used without further purification. Thin-layer chromatography (TLC) with a layer thickness of 2 mm and a grain size of 20–35 μm containing a fluorescence indicator (*λ* = 254 nm) was utilized for probing the reactions. All yields refer to isolated products after indicated purification methods. An FT-IR spectrometer (model: Magna 550, Nicolet company) was utilized for recording all FT-IR spectra and an XRD spectrometer (model: D8 Advance, Bruker company) with Cu-Kα (*λ* = 1.54 nm) line were utilized for measuring the XRD patterns. ^1^H NMR (400 MHz) and ^13^C NMR (100 MHz) spectra were run on Bruker Avance DRX in pure DMSO-*d*_6_ solvent. Chemical shifts are given in the parts per million (ppm) downfield from tetramethylsilane (TMS) as an internal reference and coupling constants are in hertz. Abbreviations used for ^1^H NMR signals are singlet (s), doublet (d), triplet (t), doublet of doublets (dd), multiplet (m). TEM imaging, BET analysis, and melting point determinations were carried out using a Zeiss EM10C device, a Mini2 device (Belsorp company; Japan), and Scientific Thermo (model, 9300; England), respectively.

### Preparation of catalyst

#### Synthesis of magnetic silica mesoporous nanoparticles (M-MSNs)

**Fig. 1:**
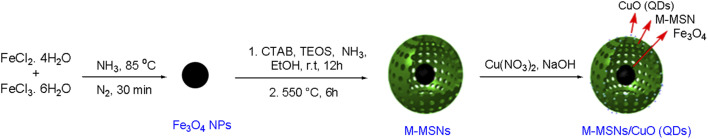
The step-wise synthesis of the M-MSNs/CuO(QDs).

In a typical synthesis, FeCl_2_·4H_2_O (0.085 gr) and FeCl_3_·6H_2_O (0.2 gr) were dissolved in 50 mL DI water at 85 °C, followed by addition of 0.6 mL NH_3_ solution (30%) and stirring under the nitrogen atmosphere at 85 °C for about 0.5 h afterwards, the resulted magnetic nanoparticles were washed with water and EtOH and dried at ambient temperature. Thereafter, 100.0 mg of iron oxide nanoparticles were suspended in 160 mL DI water, and then 0.7 gr CTAB (cetyltrimethyl ammonium bromide) was added to suspension under ultra-sonication. Afterwards, the resulting suspension was introduced to 220 mL EtOH and then 1.2 mL NH_3_ was added to the reaction media then, a (tetraethyl orthosilicate) TEOS solution (0.4 mL TEOS in 10 mL EtOH) was added and the reaction mixture was stirred for about 12 h at ambient conditions. After this time, magnetic silica mesoporous nanoparticles were collected, washed with EtOH, and calcined at 550 °C for 6 h (Fig. 1).^39^

#### Surface modification of M-MSNs with CuO QDs

To preparation of, M-MSNs/CuO(QDs) nanocomposite, initially, 100 mL NaOH (1 M) was dropped by drop added to a 0.1 M Cu(NO_3_)_2_ aqueous solution with a fixed pH of 10. Thereafter, 10 mL M-MSNs solution (100 mg mL^−1^) was introduced to the resulting solution under mild stirring. After 1 h, the M-MSNs/CuO(QDs) nanocomposite was collected and dried at 60 °C (Fig. 1).

#### General procedure for the synthesis of benzo[*a*]pyrano[2,3-*c*]phenazine derivatives using M-MSNs/CuO(QDs) under thermal conditions

An ethanolic mixture of 2-hydroxy-1,4-naphthoquinone (1.0 mmol) and *o*-phenylenediamine (1.0 mmol) was heated at 78 °C for about 5 min to produce orange-colored benzo[*a*]phenazine. Then, aromatic aldehyde (1.0 mmol), malononitrile (1.0 mmol), and M-MSNs/CuO(QDs) (0.007 gr) were introduced to the reaction media. The synthesis was followed by heating at 78 °C under mild stirring. After the completion of the synthesis process (monitored by TLC), the catalyst was separated from the mixture upon a magnetic field and the product was collected by filtration and washing with EtOH and dried.

Obtained products were identified using FTIR, ^1^HNMR and ^13^C NMR analysis method but two ^13^C NMR were not acquired due to the low solubility of the compounds (5b, 5l) hence, the elemental analysis was provided and the data pointed to both their purities and compositions.

#### Representative spectral data

##### 3-Amino-1-phenyl-1*H*-benzo[*a*]pyrano[2,3-*c*]phenazine-2-carbonitrile (5a)

Yellow solid; M.P. = 302–305 °C;^37^ FTIR(KBr, *ν*, cm^−1^): 3442–3311 (NH_2_), 3175 (C–H aromatic), 3055 (C–H aliphatic), 2185 (CN), 1657–1591 (C=C aromatic), 1156 (C–O), 841–759 (N–H); ^1^H NMR (400 MHz, DMSO-*d*_6_) (*δ*, ppm): 9.17 (d, *J* = 8.0 Hz, 1H, Ar-H), 8.41 (d, *J* = 8.0 Hz, 1H, Ar-H), 8.26–8.15 (m, 1H, Ar-H), 8.10 (t, *J* = 4.6 Hz, 1H, Ar-H), 8.01–7.85 (m, 4H, Ar-H), 7.38 (d, *J* = 6.7 Hz, 4H, Ar-H), 7.21 (t, *J* = 7.5 Hz, 2H, NH_2_), 7.08 (t, *J* = 7.5 Hz, 1H, Ar-H), 5.45 (s, 1H, CH); ^13^C NMR (100 MHz, DMSO-*d*_6_) (*δ* ppm): 159.7, 146.2, 145.3, 141.4, 140.7, 140.1, 139.6, 130.8, 130.5, 130.3, 130.1, 129.0, 128.9, 128.7, 128.4, 127.7, 126.5, 125.7, 125.1, 121.8, 120.3, 114.2, 58.2, 37.4.

##### 3-Amino-1-(4-nitrophenyl)-1*H*-benzo[*a*]pyrano[2,3-*c*]phenazine-2-carbonitrile (5b)

Yellow solid; M.P. = 278–280 °C;^37^ FTIR(KBr, *ν*, cm^−1^): 3438–3323 (NH_2_), 3202 (C–H aromatic), 3064 (C–H aliphatic), 2196 (CN), 1669–1595 (C=C aromatic), 1513–1392 (NO_2_), 1167 (C–O), 825–763 (N–H); ^1^H NMR (400 MHz, DMSO-*d*_6_) (*δ*, ppm): 9.23 (d, *J* = 8.1 Hz, 1H, Ar-H), 8.46 (d, *J* = 7.8 Hz, 1H, Ar-H), 8.26 (s, 1H, Ar-H), 8.10 (d, *J* = 8.0 Hz, 3H, Ar-H), 8.03–7.91 (m, 4H, Ar-H), 7.69 (d, *J* = 8.4 Hz, 2H, Ar-H), 7.51 (s, 2H, NH_2_), 5.61 (s, 1H, CH); anal. calcd. for C_26_H_15_N_5_O_3_: C 70.11, H 3.37, N 15.73, O 10.79%. Found: C 69.73, H 3.22, N 15.64, 10.75%.

##### 3-Amino-1-(3-nitrophenyl)-1*H*-benzo[*a*]pyrano[2,3-*c*]phenazine-2-carbonitrile (5c)

Yellow solid; M.P. = 277–279 °C;^37^ FTIR (KBr, *ν*, cm^−1^): 3424–3349 (NH_2_), 2190 (CN), 1664–1596 (C=C aromatic), 1525–1393 (NO_2_), 1163 (C–O), 762 (N–H); ^1^H NMR (400 MHz, DMSO-*d*_6_) (*δ*, ppm): 9.26 (d, *J* = 7.9 Hz, 1H, Ar-H), 8.50 (d, *J* = 8.1 Hz, 1H, Ar-H), 8.33 (s, 1H, Ar-H), 8.31 (d, *J* = 7.3 Hz, 1H, Ar-H), 8.17 (d, *J* = 7.3 Hz, 1H, Ar-H), 8.04 (s, 2H, NH_2_), 8.01–7.89 (m, 4H, Ar-H), 7.56 (s, 3H, Ar-H), 5.73 (s, 1H, CH); ^13^C NMR (100 MHz, DMSO-*d*_6_) (*δ* ppm): 159.8, 147.5, 147.3, 145.9, 141.1, 140.0, 139.8, 139.4, 134.6, 130.5, 129.8, 128.9, 128.3, 125.2, 124.5, 122.5, 122.0, 121.6, 119.9, 112.1, 56.8, 37.3.

##### 3-Amino-1-(4-bromophenyl)-1*H*-benzo[*a*]pyrano[2,3-*c*]phenazine-2-carbonitrile (5d)

Yellow solid; M.P. = 281–284 °C;^37^ FTIR (KBr, *ν*, cm^−1^): 3455–3318 (NH_2_), 2191 (CN), 1666–1591 (C=C aromatic), 1160 (C–O), 834–760 (N–H), 760 (C–Br); ^1^H NMR (400 MHz, DMSO-*d*_6_) (*δ*, ppm): 9.27 (d, *J* = 7.9 Hz, 1H, Ar-H), 8.47 (d, *J* = 7.9 Hz, 1H, Ar-H), 8.31 (s, 1H, Ar-H), 8.19 (d, *J* = 7.6 Hz, 1H, Ar-H), 8.08–7.90 (m, 4H, Ar-H), 7.43 (d, *J* = 10.7 Hz, 4H, Ar-H), 7.38 (d, *J* = 8.9 Hz, 2H, NH_2_), 5.53 (s, 1H, CH); ^13^C NMR (100 MHz, DMSO-*d*_6_) (*δ* ppm): 159.7, 157.0, 146.1, 144.6, 141.4, 141.3, 139.9, 131.1, 130.4, 129.9, 129.0, 128.6, 125.5, 124.8, 122.1, 119.5, 113.0, 57.4, 37.0.

##### 3-Amino-1-(4-chlorophenyl)-1*H*-benzo[*a*]pyrano[2,3-*c*]phenazine-2-carbonitrile (5e)

Yellow solid; M.P. = 287–290 °C;^37^ FTIR(KBr) *ν* (cm^−1^): 3455–3318 (NH_2_), 2191 (CN), 1666–1591 (C=C aromatic), 1160 (C–O), 834–760 (N–H), 760 (C–Cl); ^1^H NMR (400 MHz, DMSO-*d*_6_) (*δ* ppm): 9.27 (d, *J* = 7.9 Hz, 1H, Ar-H), 8.47 (d, *J* = 7.9 Hz, 1H, Ar-H), 8.31 (s, 1H, Ar-H), 8.19 (d, *J* = 7.6 Hz, 1H, Ar-H), 8.05–7.93 (m, 4H, Ar-H), 7.43 (d, *J* = 10.7 Hz, 4H, Ar-H), 7.38 (d, *J* = 8.9 Hz, 2H, NH_2_), 5.53 (s, 1H, CH); ^13^C NMR (100 MHz, DMSO-*d*_6_) (*δ* ppm): 160.1, 152.2, 144.4, 140.7, 140.2, 131.0, 130.7, 130.5, 130.3, 130.0, 129.5, 129.1, 128.6, 128.3, 125.8, 125.0, 122.8, 120.1, 113.4, 57.5.

##### 3-Amino-1-(*p*-tolyl)-1*H*-benzo[*a*]pyrano[2,3-*c*]phenazine-2-carbonitrile (5f)

Yellow solid; M.P. = 294–297 °C;^37^ FTIR(KBr, *ν*, cm^−1^): 3440–3311 (NH_2_), 3179 (C–H aromatic), 3052 (C–H aliphatic), 2187 (CN), 1659–1599 (C=C aromatic), 1160 (C–O), 833–759 (N–H); ^1^H NMR (400 MHz, DMSO-*d*_6_) (*δ* ppm): 9.26 (d, *J* = 8.0 Hz, 1H, Ar-H), 8.46 (d, *J* = 7.9 Hz, 1H, Ar-H), 8.30 (s, 1H, Ar-H), 8.19 (s, 1H, Ar-H), 8.05–7.94 (m, 4H, Ar-H), 7.41–7.29 (m, 4H, Ar-H), 7.03 (d, *J* = 7.8 Hz, 2H, NH_2_), 5.49 (s, 1H, CH), 2.15 (s, 3H, CH_3_); ^13^C NMR (100 MHz, DMSO-*d*_6_) (*δ* ppm): 159.7, 145.9, 142.3, 141.4, 140.5, 139.9, 139.6, 135.5, 130.3, 130.0, 128.8, 127.4, 125.5, 124.7, 121.9, 120.2, 113.9, 58.1, 36.9, 20.4.

##### 3-Amino-1-(2-chlorophenyl)-1*H*-benzo[*a*]pyrano[2,3-*c*]phenazine-2-carbonitrile (5g)

Yellow solid; M.P. = 299–303 °C;^37^ FTIR (KBr, *ν*, cm^−1^): 3463–3312 (NH_2_), 3171 (CH aromatic), 3059 (CH aliphatic), 2189 (CN), 1658–1586 (C=C aromatic), 1161 (C–O), 754 (N–H), 754 (C–Cl); ^1^H NMR (400 MHz, DMSO-*d*_6_) (*δ* ppm): 9.28 (s, 1H, Ar-H), 8.50 (s, 1H, Ar-H), 8.29 (s, 1H, Ar-H), 8.06–7.93 (m, 5H, Ar-H), 7.43 (s, 1H, Ar-H), 7.36 (s, 2H, NH_2_), 7.23 (s, 1H, Ar-H), 7.12 (s, 2H, Ar-H), 6.00 (s, 1H, CH); ^13^C NMR (100 MHz, DMSO-*d*_6_) (*δ* ppm): 170.1, 159.1, 146.3, 142.3, 141.2, 140.3, 140.0, 139.6, 132.0, 130.6, 130.3, 130.1, 130.0, 129.2, 129.1, 129.0, 128.3, 128.0, 127.1, 125.2, 124.5, 122.1, 119.2, 112.7, 56.6, 36.5.

##### 3-Amino-1-(4-methoxyphenyl)-1*H*-benzo[*a*]pyrano[2,3-*c*]phenazine-2-carbonitrile (5h)

Yellow solid; M.P. = 271–273 °C;^37^ FTIR (KBr, *ν*, cm^−1^): 3432–3319 (NH_2_), 3197 (CH aromatic), 3047 (CH aliphatic), 2194 (CN), 1666–1598 (C=C aromatic), 1165 (C–O), 834–761 (N–H); ^1^H NMR (400 MHz, DMSO-*d*_6_) (*δ* ppm): 9.26 (d, *J* = 8.0 Hz, 1H, Ar-H), 8.46 (d, *J* = 7.9 Hz, 1H, Ar-H), 8.30 (s, 1H, Ar-H), 8.19 (s, 1H, Ar-H), 8.04–7.95 (m, 4H, Ar-H), 7.39–7.29 (m, 4H, Ar-H), 7.03 (d, *J* = 7.8 Hz, 2H, NH_2_), 5.49 (s, 1H, CH), 3.61 (s, 3H, CH_3_); ^13^C NMR (100 MHz, DMSO-*d*_6_) (*δ* ppm): 159.7, 157.8, 145.9, 140.7, 140.0, 137.3, 130.5, 130.4, 130.2, 129.0, 128.6, 125.6, 124.9, 122.1, 120.2, 114.2, 113.6, 58.14, 54.86, 36.5.

##### 3-Amino-1-(3-methoxyphenyl)-1*H*-benzo[*a*]pyrano[2,3-*c*]phenazine-2-carbonitrile (5i)

Yellow solid; M.P. = 237–242 °C;^40^ FTIR(KBr, *ν*, cm^−1^): 3420 (NH_2_), 2192 (CN), 1664–1602 (C=C aromatic), 1161 (C–O), 754 (N–H); ^1^H NMR (400 MHz, DMSO-*d*_6_) (*δ* ppm): 9.23 (s, 1H, Ar-H), 8.44 (s, 1H, Ar-H), 8.28 (s, 1H, Ar-H), 8.18 (s, 1H, Ar-H), 8.02–7.91 (m, 4H, Ar-H), 7.38 (s, 2H, NH_2_), 7.13 (s, 1H, Ar-H), 7.02 (s, 1H, Ar-H), 6.92 (s, 1H, Ar-H), 6.67 (s, 1H, Ar-H), 5.50 (s, 1H, CH), 3.67 (d, *J* = 3.5 Hz, 3H, CH_3_); ^13^C NMR (100 MHz, DMSO-*d*_6_) (*δ* ppm): 159.9, 159.0, 146.7, 146.0, 141.4, 140.5, 139.9, 130.6, 130.1, 129.4, 129.02, 128.6, 125.5, 124.7, 122.0, 120.2, 119.6, 113.8, 111.5, 57.7, 54.8, 37.2.

##### 3-Amino-1-(3-bromophenyl)-1*H*-benzo[*a*]pyrano[2,3-*c*]phenazine-2-carbonitrile (5j)

Brown solid; M.P. = 267–269 °C;^37^ FTIR (KBr, *ν*, cm^−1^): 3442–3319 (NH_2_), 2195 (CN), 1659–1591 (C=C aromatic), 1159 (C–O), 854–759 (N–H); ^1^H NMR (400 MHz, DMSO-*d*_6_) (*δ* ppm): 9.24 (d, *J* = 8.0 Hz, 1H, Ar-H), 8.45 (s, 1H, Ar-H), 8.28 (s, 1H, Ar-H), 8.15 (s, 1H, Ar-H), 8.01–7.91 (m, 4H, Ar-H), 7.62 (s, 1H, Ar-H), 7.50–7.43 (m, 2H, Ar-H), 7.30 (s, 2H, NH_2_), 7.24–7.17 (m, 1H, Ar-H), 5.52 (s, 1H, CH); ^13^C NMR (100 MHz, DMSO-*d*_6_) (*δ* ppm): 159.8, 147.9, 146.1, 141.3, 140.3, 139.9, 139.6, 130.6, 130.5, 130.3, 129.4, 129.0, 128.4, 126.8, 125.4, 122.0, 121.4, 120.0, 112.8, 57.3, 37.2.

##### 1,1′-([1,1′-biphenyl]-4,4′-diyl)bis(3-amino-1*H*-benzo[*a*]pyrano[2,3-*c*]phenazine-2-carbonitrile) (5k)

Yellow solid; M.P. = 287–289 °C;^41^ FTIR(KBr, *ν*, cm^−1^): 3430 (NH_2_), 2190 (CN), 1663–1594 (C=C aromatic), 1158 (C–O), 823–759 (N–H); ^1^H NMR (400 MHz, DMSO-*d*_6_) (*δ* ppm): 9.20 (d, *J* = 7.7 Hz, 2H, Ar-H), 8.44 (d, *J* = 8.1 Hz, 2H, Ar-H), 8.38 (s, 2H, Ar-H), 8.24 (s, 2H, Ar-H), 8.11 (s, 2H, Ar-H), 8.05–7.87 (m, 8H, Ar-H, NH_2_), 7.83 (d, *J* = 8.2 Hz, 2H, Ar-H), 7.65 (d, *J* = 8.4 Hz, 2H, Ar-H), 7.51 (s, 2H, Ar-H), 5.54 (s, 2H, CH); ^13^C NMR (100 MHz, DMSO-*d*_6_) (*δ* ppm): 161.44, 160.21, 152.69, 146.86, 141.86, 140.90, 131.35, 131.16, 130.90, 130.70, 130.17, 129.63, 129.49, 129.39, 129.09, 125.95, 125.26, 122.68, 120.41, 114.76, 112.81, 80.89, 57.46.

##### 3-Amino-1-(2,4-dichlorophenyl)-1*H*-benzo[*a*]pyrano[2,3-*c*]phenazine-2-carbonitrile (5l)

Brown solid; M.P. = 308–311 °C;^40^ FTIR (KBr, *ν*, cm^−1^): 3471–3312 (NH_2_), 2185 (CN), 1657–1592 (C=C aromatic), 1163 (C–O), 847–760 (N–H), 760 (C–Cl); ^1^H NMR (400 MHz, DMSO-*d*_6_) (*δ* ppm): 9.23 (s, 1H, Ar-H), 8.44 (s, 1H, Ar-H), 8.26 (s, 1H, Ar-H), 8.06–7.85 (m, 5H, Ar-H), 7.64–7.55 (m, 1H, Ar-H), 7.42 (s, 2H, NH_2_), 7.22 (s, 1H, Ar-H), 7.17 (s, 1H, Ar-H), 5.91 (s, 1H, CH); anal. calcd. for C_26_H_14_Cl_2_N_4_O: C 66.52, H 2.99, N 11.94, O 3.41, Cl 15.14%. Found: C 66.48, H 2.86, N 11.81, O 3.25%.

## Results and discussion

### Characterization of M-MSNs/CuO(QDs)

The as-prepared nanocatalyst was characterized by FT-IR, EDX, BET & BJH, XRD, VSM, SEM, and TEM techniques for their functionality, elemental composition, surface properties, crystalline structure, magnetic properties, size, and morphological characteristics.

**Fig. 2:**
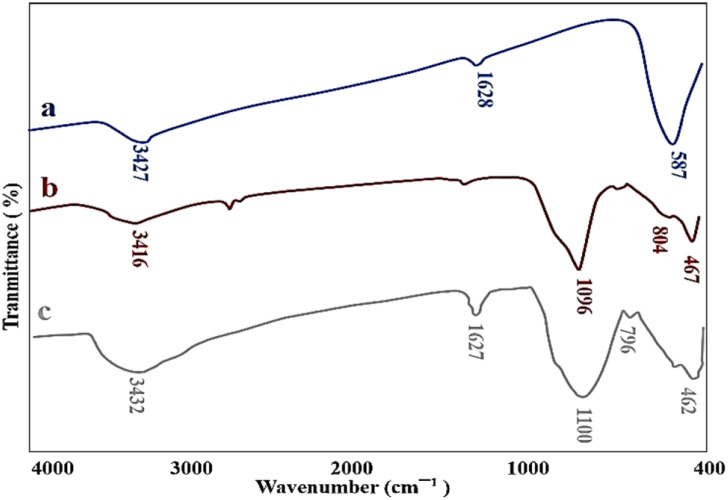
FT-IR spectra of (a) Fe_3_O_4,_ (b) M-MSNs and (d) M-MSNs/CuO(QDs).

The FT-IR analysis was performed to prove the successful synthesis of M-MSNs/CuO nanocatalyst. In this regard, the FT-IR spectra of MNPs, M-MSNs, and M-MSNs/CuO(QDs) were recorded (Fig. 2a). In spectrum a (MNPs), the peak at 3427 cm^−1^ is related to the –OH group and the vibrational band at 1628 cm^−1^ is assigned to the adsorbed water on the surface of the MNPs.^41^ Moreover, the main peak positioned at 587 cm^−1^ is contributed to the Fe–O vibration.^42^ Regarding the M-MSNs spectrum, the bands at 3416 cm^−1^, 1096 cm^−1^, 804 cm^−1^, and 467 cm^−1^ are related to the –OH vibrations, Si–O–Si groups of silica, Si–O of Si–OH bonds stretching vibration and Si–OH bending in the structure of the as-prepared M-MSNs, in order^43^ (Fig. 2b). Moreover, after modification of M-MSNs with CuO QDs, the characteristic peak at 462 and 525 are from stretching mode of CuO(QDs) (in addition to the main peaks of M-MSNs). Obtained results confirmed the successful synthesis of M-MSNs/CuO(QDs) nanocatalyst (Fig. 2c).

**Fig. 3:**
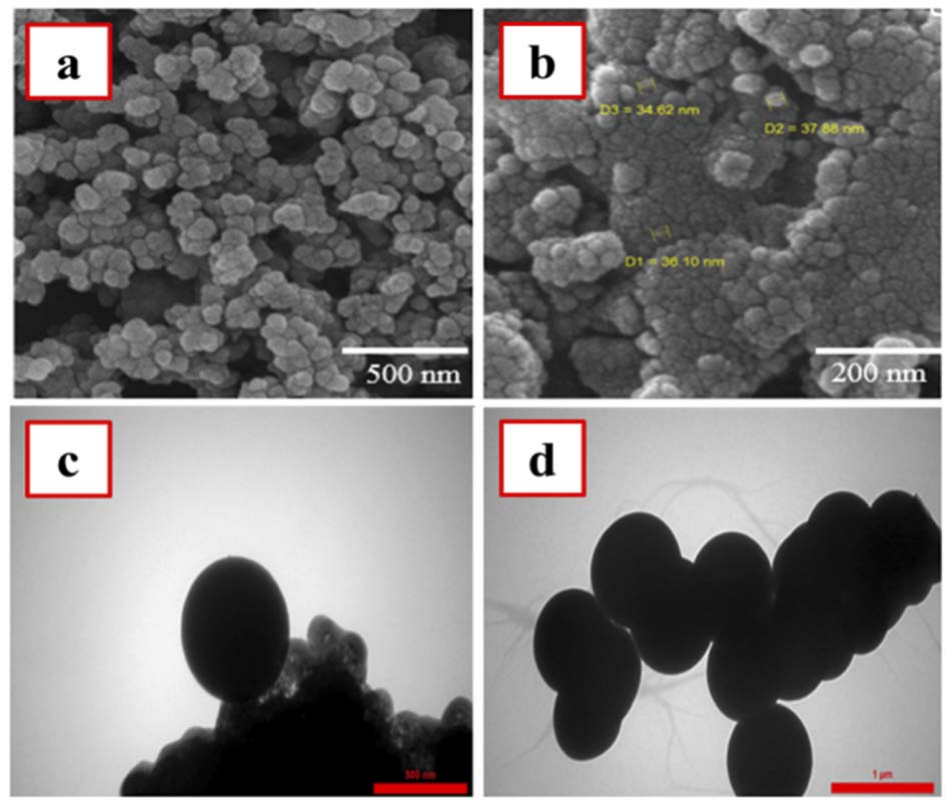
SEM and TEM images of M-MSNs (a) and (c) and M-MSNs/CuO(QDs) (b) and (d).

The size and morphology of the as-prepared nanomaterials were investigated by SEM and TEM imaging methods. Based on the SEM images (Fig. 3a and b), the morphological properties of M-MSN nanoparticles were not changed after their modification with CuO QDs. Moreover, these images revealed spherical and uniform particles for the as-prepared M-MSNs/CuO(QDs) nanocatalyst with a narrow size distribution over 34.6–38.0 nm. However, to explore more precisely the spherical morphology of the as-prepared M-MSNs/CuO nanocatalyst, the TEM images of both M-MSNs and M-MSNs/CuO(QDs) were recorded (Fig. 3c and d). The results showed that both M-MSNs and M-MSNs/CuO(QDs) nanocatalysts have uniform and spherical particles, in agreement to the results of the SEM imaging method.

**Fig. 4:**
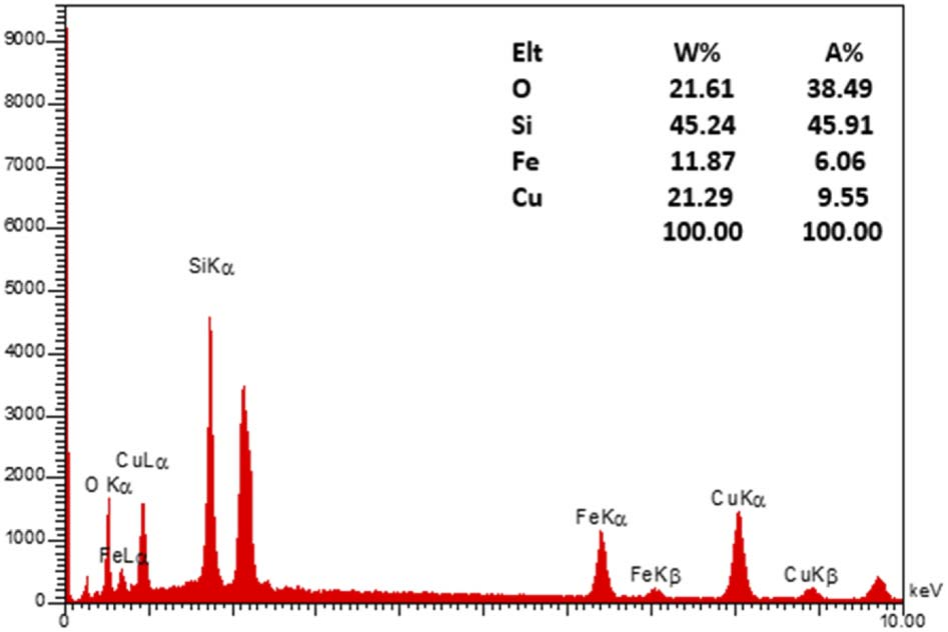
EDX spectrum of M-MSNs/CuO(QDs).

**Fig. 5:**
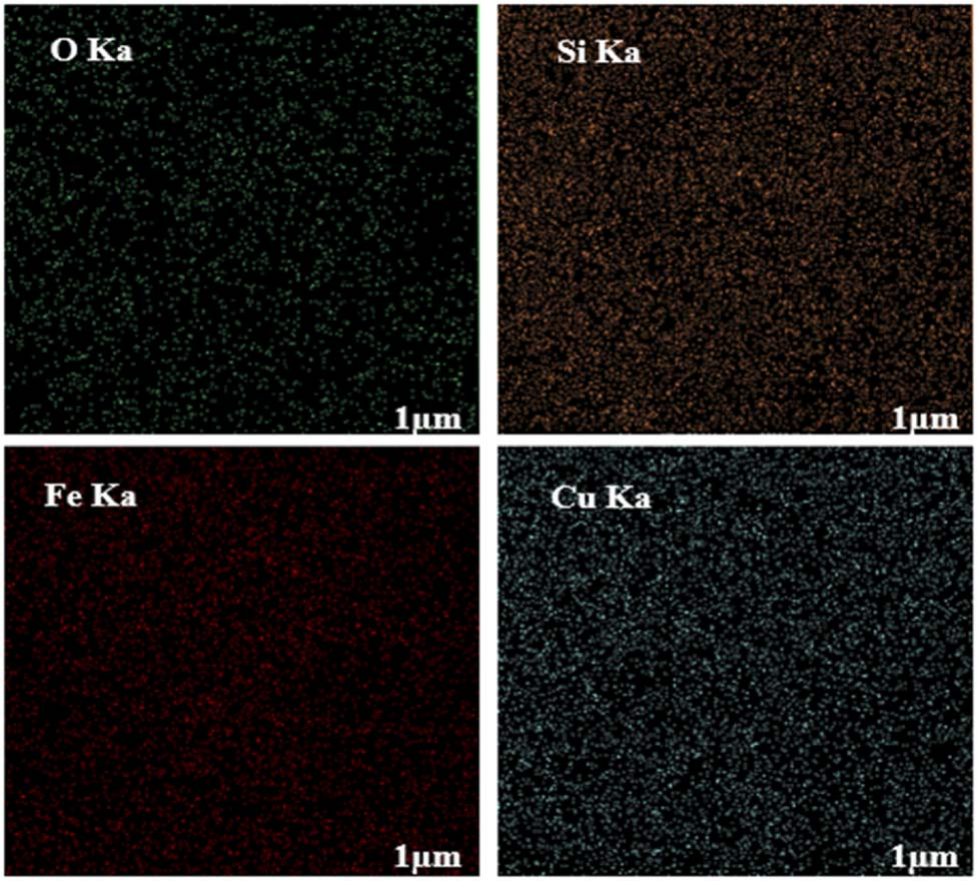
Elemental EDX mapping of O, Fe, Si, and Cu in M-MSNs/CuO(QDs).

To evaluate the elemental composition of the as-prepared nanocatalyst, the EDX analysis was also performed (Fig. 4). The lines of Fe, O, Si, and Cu are obviously observed in the EDX pattern of the nanocatalyst, revealing the successful synthesis of the M-MSNs/CuO(QDs) upon the developed method. Moreover, the mapping analysis exhibited the presence of O, Fe, Si, and Cu elements in the structure of the M-MSNs/CuO(QDs), regarding to the EDX results (Fig. 5).

**Fig. 6:**
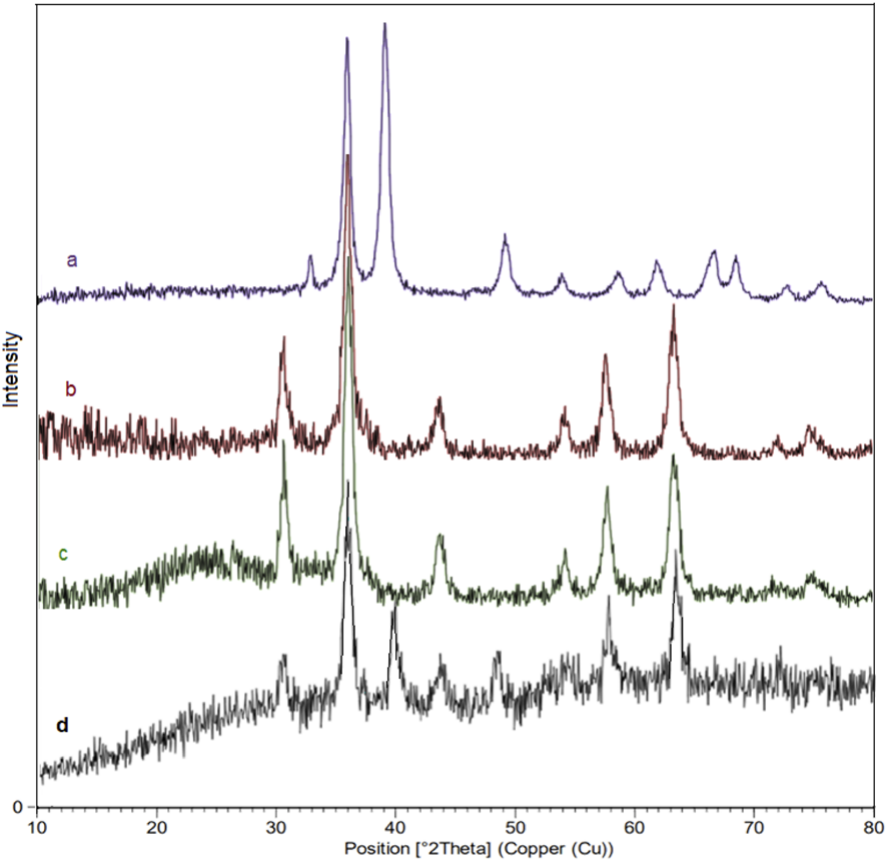
The X-ray diffraction (XRD) pattern of (a) CuO QDs, (b) Fe_3_O_4,_ (c) M-MSNs and (d) M-MSNs/CuO(QDs).

Moreover, to evaluate the crystalline properties of the nanocatalyst, the XRD patterns of CuO(QDs), MNPs, M-MSNs, and M-MSNs/CuO(QDs) were recorded (Fig. 6). The XRD pattern of CuO(QDs) (Fig. 6a) show the peaks at 2*θ* = 32.58°, 35.47°, 38.97° and 48.74° that are assigned to (110), (002), (200), (202), and plane orientation of CuO (JCPDS 80-1268). Regarding to spectrum of Fe_3_O_4_ NPs (Fig. 6b), reflections are observed at 2*θ* = 30°, 35°, 43°, 53°, 57°, and 63° belonging to the (220), (311), (400), (422), (511), and (440) plates respectively, revealed the cubic spinel crystalline structure of Fe_3_O_4_ which are in agreement with the standard data (JCPDS card no. 19-629).^44^ However, after the synthesis of magnetic silica mesoporous nanoparticles, new broadband was observed at 2*θ* of 20–30° related to SiO_2_ amorphous structure (spectrum c). Moreover, in the XRD pattern of M-MSNs/CuO(QDs) nanocatalyst (spectrum d), the peaks at 30.36°, 35.76°, 43.47°, 57.51°, 63.16° are assigned to (022), (113), (004), (115), and (044) plates of CuO, in turn. In addition, the main peaks of M-MSNs were saved after its modification with CuO QDs, exhibiting that the crystalline structure of M-MSNs was saved during the synthesis of M-MSNs/CuO(QDs).

**Fig. 7:**
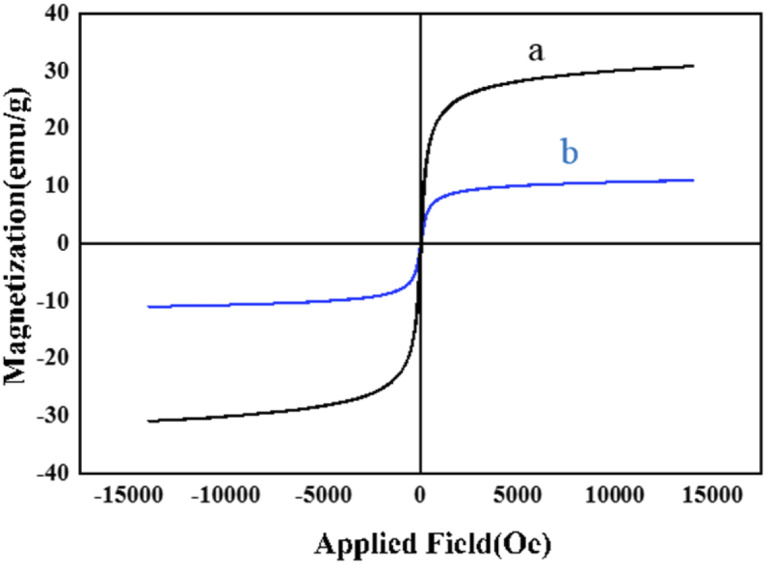
Magnetic hysteresis curves of (a) Fe_3_O_4_, and (b) M-MSNs/CuO(QDs).

To investigate the magnetic properties of the as-prepared nanocatalysts as one of the most important factors which may affect the reusability and handling of a catalyst, the VSM analysis was carried out for both bare MNPs and M-MSNs/CuO(QDs) at 25 °C (Fig. 7). The results showed that the magnetic properties of nanocatalyst was significantly lower than that of the bare MNPs. This is related to the presence of a silica shell in the structure of the M-MSNs/CuO(QDs), leading to a decrease in the magnetic properties of the MNPs.

**Fig. 8:**
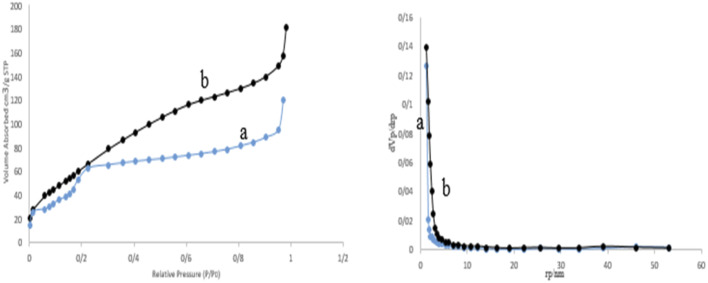
BET results of M-MSNs (a) & M-MSNs/CuO(QDs) (b).

The porosity and surface area of the prepared M-MSNs/CuO(QDs) nanocatalyst was calculated by the BET analysis (Fig. 8). Based on this analysis, the surface area of M-MSNs and M-MSNs/CuO(QDs) was estimated at about 284.68 m^2^ g^−1^ and 354.41 m^2^ g^−1^, respectively. The decrease in the surface area of the M-MSN nanoparticles after modification with CuO QDs can be attributed to the occupation of the holes of M-MSNs with the QDs during the synthesis process.

After the successful synthesis and characterization of M-MSNs/CuO (QDs), we aimed to investigate its efficiency as a new, reusable and nanocatalyst for the synthesis of benzo[*a*]pyrano-[2,3-*c*]phenazines dyes.

### Screening the conditions for the synthesis of the benzo[*a*]pyrano-[2,3-*c*]phenazines in the presence of M-MSNs/CuO(QDs)

To optimize the reaction conditions, the reaction between 2-hydroxy-1,4-naphthoquinone (1 mmol), benzene-1,2-diamine (1 mmol), 4-nitrobenzaldehyde (1 mmol), and malononitrile (1 mmol) to the corresponding product was selected as a model reaction and various conditions including reaction media, temperature, and amount of the catalyst were examined. The results are summarized in Table 1. To find the optimal reaction conditions, various solvents were used in model reaction in the presence of M-MSNs/CuO (ODs) under conventional heating conditions (Table 1, entries 1–4). The best results were obtained in the EtOH (entry 4). Eventually, ethanol was preferred as the solvent from the viewpoint of both yield and green chemistry principles. It should be mentioned that, in the presence of H_2_O as the only solvent, the reaction efficiency was negligible (entry 1). In the following, the temperature screening indicated that decreasing the reaction temperature from reflux temperature to 60 °C decreased the product yield (entry 6). Hence, 78 °C was selected as optimal temperature. Finally, model reaction was carried out using different amounts of M-MSNs/CuO(QDs) (entries 8–10) and the best results were obtained when 7 mg of M-MSNs/CuO(QDs) was used (entry 4). Increasing the amount of M-MSNs/CuO(QDs) to more than 7 mg did not improve the yield and the reaction time substantially (entry 10). The use of less than 7 mg of M-MSNs/CuO(QDs) led to lower yields (entries 8,9). Thereupon, the optimized conditions were found to be using EtOH as a solvent, in the presence of 7 mg (0.0018 mol%) of catalyst at 78 °C.

**Table 1:** Optimization of the reaction conditions for the synthesis of benzo[*a*]pyrano-[2,3-*c*]phenazines^a^

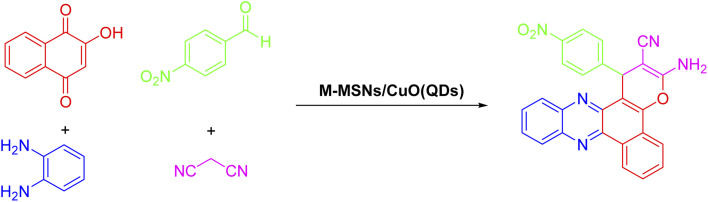					
Entry	Catalyst (g)	Solvent	Temp. (°C)	Time (min)	Yield^c^ (%)
1	0.007^b^	H_2_O	Reflux	85	Trace
2	0.007	EtOH/H_2_O	Reflux	80	65
3	0.007	Toluene	Reflux	70	30
4	0.007	EtOH	78	70	95
5	0.007	EtOH	r.t.	130	25
6	0.007	EtOH	60	95	55
7	0.007	EtOH	70	80	68
8	0.005	EtOH	78	110	32
9	0.006	EtOH	78	80	65
12	0.008	EtOH	78	70	95

a Reaction conditions: 2-hydroxy-1,4-naphthoquinone (1 mmol), benzene-1,2-diamine (1 mmol), 4-nitrobenzaldehyde (1 mmol), malononitrile (1 mmol), M-MSNs/CuO(QDs), solvent (2 mL).

b Mol% of catalyst was 0.0018, 0.0016, 0.0013 and 0.0021 for 7, 6, 5 and 8 mg of catalyst respectively.

c Isolated yield.

Encouraged by the initial success in the production of 3-amino-1-(4-nitrophenyl)-1*H*-benzo[*a*]pyrano[2,3-*c*]phenazine-2-carbonitrile (5b) *via* the multicomponent reaction strategy, to show the general scope and versatility of this strategy in the preparation of benzo[*a*]pyrano-[2,3-*c*]phenazine derivatives, different aromatic aldehydes containing electron-releasing and electron-withdrawing substituents were examined under optimized conditions. Excitingly, the corresponding benzo[*a*]pyrano-[2,3-*c*]phenazine derivatives were successfully and smoothly obtained, and the results are listed in Table 2 (5a–5l). As shown in Table 2, both electron-releasing groups and electron-withdrawing groups on aromatic aldehydes structure presented well to excellent yield with a slightly better result for electron-withdrawing groups.

**Table 2:** Synthesis of benzo[*a*]pyrano-[2,3-*c*]phenazine derivatives catalyzed by M-MSNs/CuO(QDs)^a^

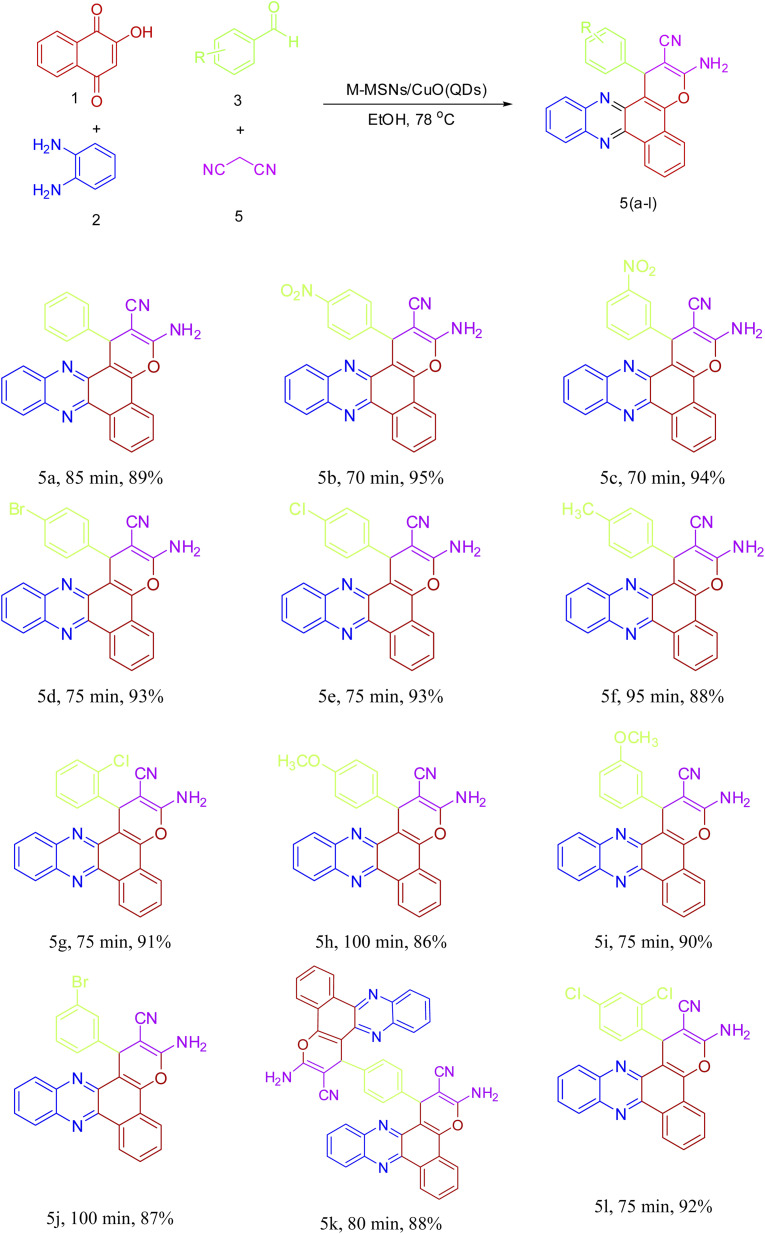

a Reaction conditions: 2-hydroxy-1,4-naphthoquinone (1 mmol), benzene-1,2-diamine (1 mmol), different aromatic aldehydes (1 mmol), malononitrile (1 mmol), M-MSNs/CuO QDs (7 mg/0.0018 mol%), EtOH (2 mL), 78 °C.

### Mechanism of the catalytic reaction

**Scheme 2:**
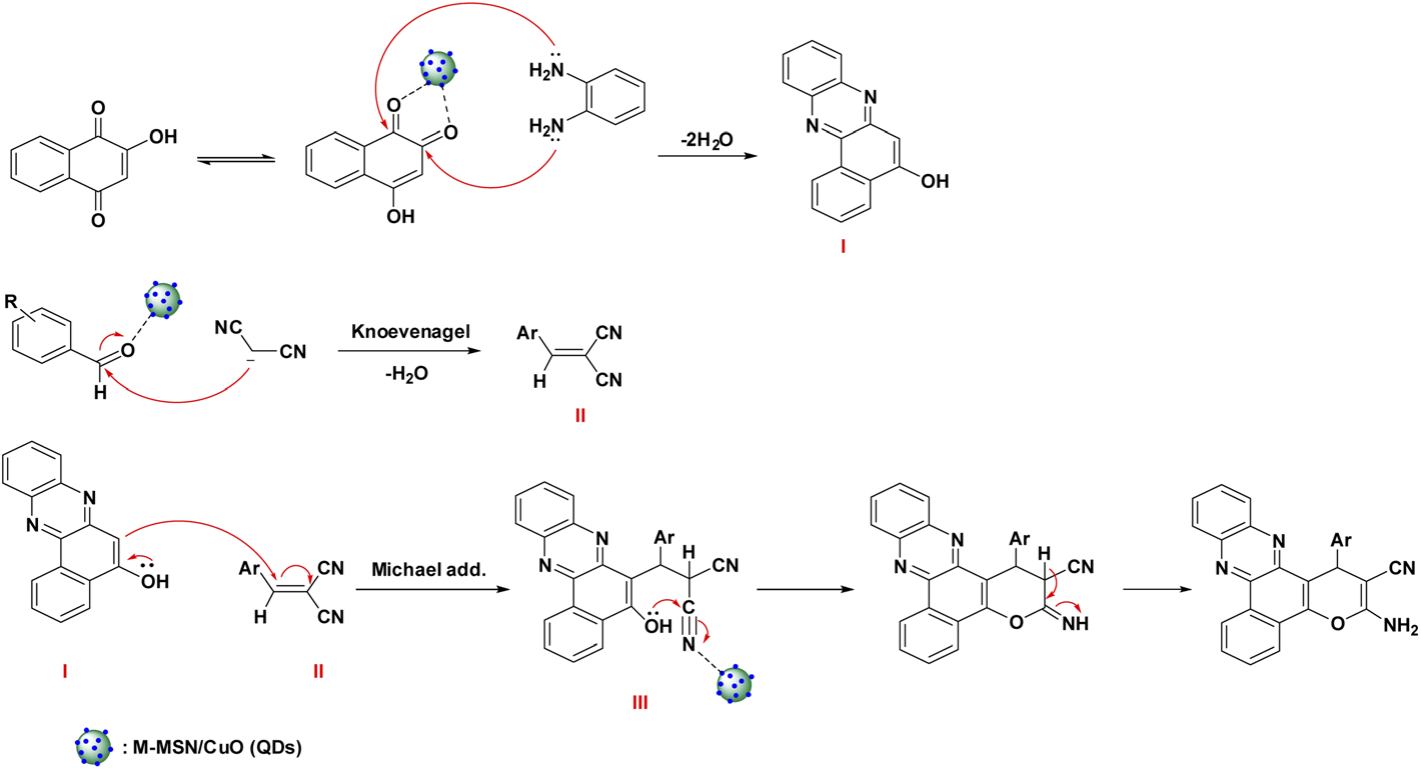
Mechanism of the catalytic synthesis of benzo[*a*]pyrano[2,3-*c*]phenazine derivatives catalyzed by M-MSNs/CuO(QDs).

The possible mechanism of the catalytic synthesis of benzo[*a*]pyrano[2,3-*c*]phenazine derivatives catalyzed by M-MSNs/CuO(QDs) was proposed to gain a deeper understanding of the developed catalytic process. The schematic of the reaction mechanism is shown in Scheme 2. As can be seen, initially, 2-hydroxy-1,4-naphthoquinone was converted to its tautomerized form. Afterwards, from the condensation reaction of *o*-phenylenediamine (OPD) and 2-hydroxy-1,4-naphthoquinone in the presence of M-MSNs/CuO(QDs), intermediate (I) was formed. Besides, the intermediate (II) was simultaneously produced by Knoevenagel condensation of malononitrile and the carbonyl group of (activated) aldehyde. After that, intermediate (I) and (II) interacted *via* Michael addition reaction in the presence of catalyst to produce the intermediate (III). Thereafter, the oxygen attacked to the carbon of the nitrile group, follow by imine-enamine tautomerization, lead to producing the final product.

### Reusability of catalyst

**Fig. 9:**
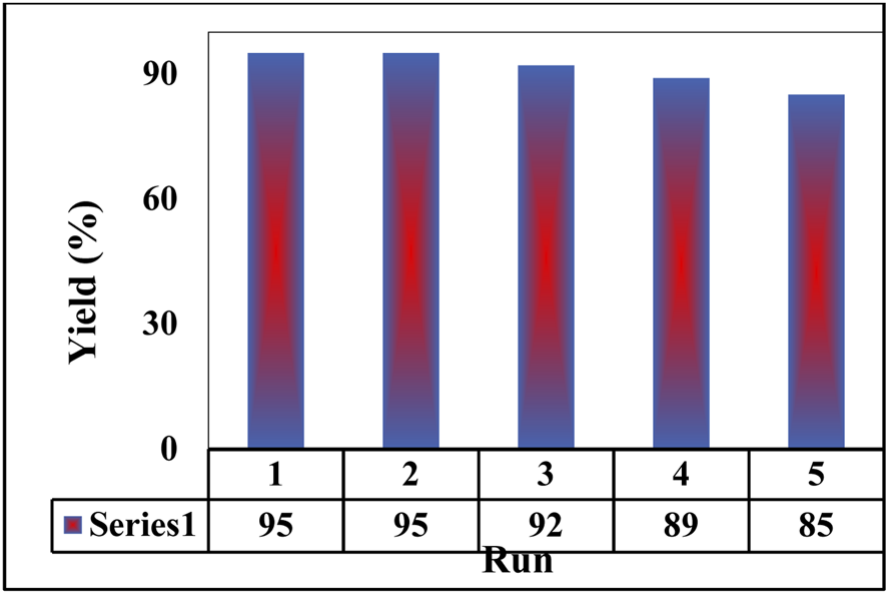
Reusability of the prepared M-MSNs/CuO(QDs) nanocatalyst.

As reported in the literatures, the reusability of a nano catalyst is an index for its cycling stability. Hence, the reusability of the prepared M-MSNs/CuO(QDs) nanocomposite was checked as one of the most important features of a catalyst. To do this, after each reaction of 2-hydroxy-1,4-naphthoquinone (1 mmol), benzene-1,2-diamine (1 mmol), 4-nitrobenzaldehyde (1 mmol), malononitrile (1 mmol) in the presence of 7 mg of M-MSNs/CuO(QDs), the catalyst was separated from reaction media using external magnetic field, washed with EtOH/water, dried and reused for the next reaction. The results in Fig. 9 indicated that after five reuses, the reaction yield was decreased (after 5 cycles) about 10% which reveals the high cycling stability and reusability of the prepared nanocatalyst.

To compare the catalytic performances of the prepared nanocatalyst with the reported ones, the reaction between *o*-phenylenediamine (OPD), 4-nitrobenzaldehyde, malononitrile, and 2-hydroxy-1,4-naphthoquinone was performed in EtOH at 78 °C using the M-MSNs/CuO(QDs) as catalyst. The results were collected in Table 3. As can be seen, the reaction time in the presence of M-MSNs/CuO(QDs) was found to be lower than that of some catalysts for instance DABCO, Gl.ACOH, oxalic acid, Et_3_N. It is mentionable that the reaction yield was also comparable with the other reported catalyst. Based on these results, it can be concluded that the prepared M-MSNs/CuO(QDs) nanocatalyst showed excellent catalytic performances as well as greener reaction conditions compared to some of the reported catalysts.

**Table 3:** Comparison of the catalytic performances of the as-prepared nanocatalyst with the reported ones

Entry	Catalyst	Condition	Time (min)	Yield (%)	Ref.
1	AcOH	MW, r.t.	9	92	34
2	Gl.AcOH	70 °C	180	85	45
3	Oxalic acid	EtOH/H_2_O, reflux	120	92	35
4	Et_3_N	MeCN/EtOH, r.t.	1440	86	38
5	DABCO	EtOH, reflux	600	60	24
6	Pyridine	EtOH, r.t.	45	90	36
7	β-Cyclodextrin	EtOH/H_2_O, 70 °C	50	92	37
8	M-MSNs	EtOH, 78 °C	70	67	—
9	CuO(QDs)	EtOH, 78 °C	70	81	—
10	M-MSNs/CuO(QDs)	EtOH, 78 °C	70	95	This work

## Conclusion

High throughput and reusable copper oxide quantum dots-modified magnetic silica mesoporous core–shell nanocatalysts (M-MSNs/CuO QDs) was synthesized and characterized by different characterization methods, including FT-IR, EDX, XRD, BET&BJH, TEM, SEM, VSM, and mapping analysis. The prepared magnetic core–shell nanocatalyst was then applied for developing a green, simple, efficient, and straightforward nanocatalytic process for the synthesis of benzo[*a*]pyrano[2,3-*c*]phenazine derivatives under mild thermal conditions. The factors affecting the reaction yield and catalytic performances of the developed nanocatalyst were optimized. The reaction yield was obtained over 86–95% for different derivatives under optimal reaction conditions. The prepared magnetic core–shell nanocatalyst showed high cycling stability and its catalytic efficiency was saved for at least 5 operational times. Moreover, the developed nanocatalyst presents very better performances toward the synthesis of benzo[*a*]pyrano[2,3-*c*]phenazine derivatives than the reported ones.

## Data availability

The data that supports the findings of this study are available in the ESI† of this article.

## Conflicts of interest

There are no conflicts to declare.

## Supplementary Material

RA-012-D2RA03887K-s001
