# Supplementary material for: Introducing a green nanocatalytic process toward the synthesis of benzo[a]pyrano-[2,3-c]phenazines utilizing copper oxide quantum dot-modified core–shell magnetic mesoporous silica nanoparticles as high throughput and reusable nanocatalysts
Source: RSC Adv. 2022 Sep 5;12(39):25194–203. doi: 10.1039/d2ra03887k (PMC9443676; doi:10.1039/d2ra03887k)
Supplement: RA-012-D2RA03887K-s001 [file RA-012-D2RA03887K-s001.pdf]

Electronic Supplementary Material (ESI) for RSC Advances.
This journal is © The Royal Society of Chemistry 2022

Introducing a green nanocatalytic process toward the synthesis of benzo[*a*]pyrano-[2,3-*c*] phenazines utilizing copper oxide quantum dots-modified core-shell magnetic silica mesoporous nanocatalysts as high throughput and reusable nanocatalysts

Mohadeseh Dehnavian, Abdulhamid Dehghani, Leila Moradi*

Department of Organic Chemistry, Faculty of Chemistry, University of Kashan, Kashan, Iran, P.O. Box 8731753153, +983155912336

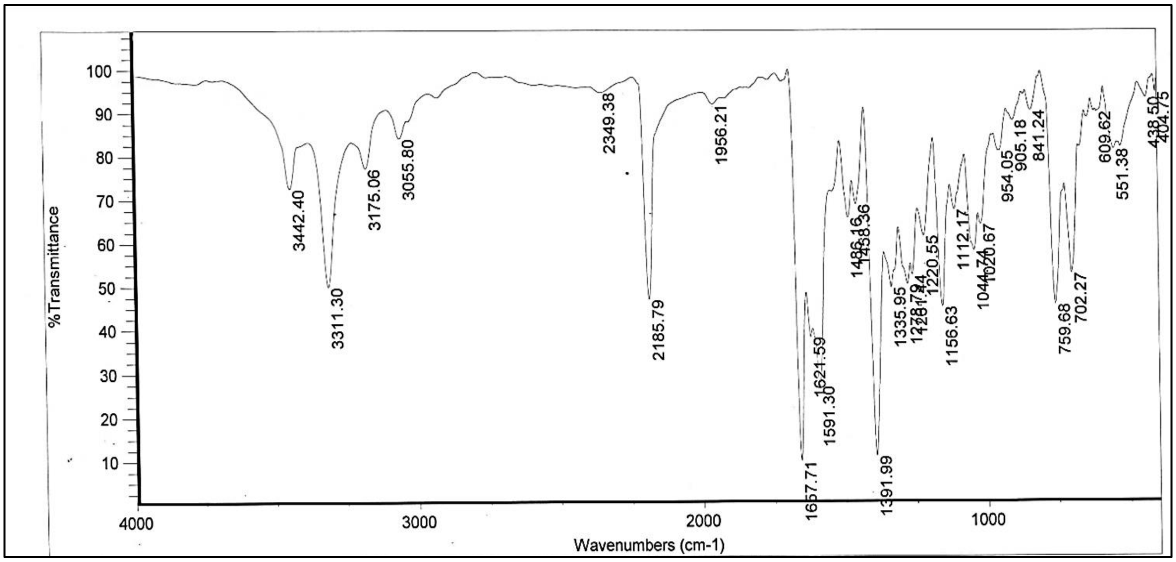

FTIR of 5a

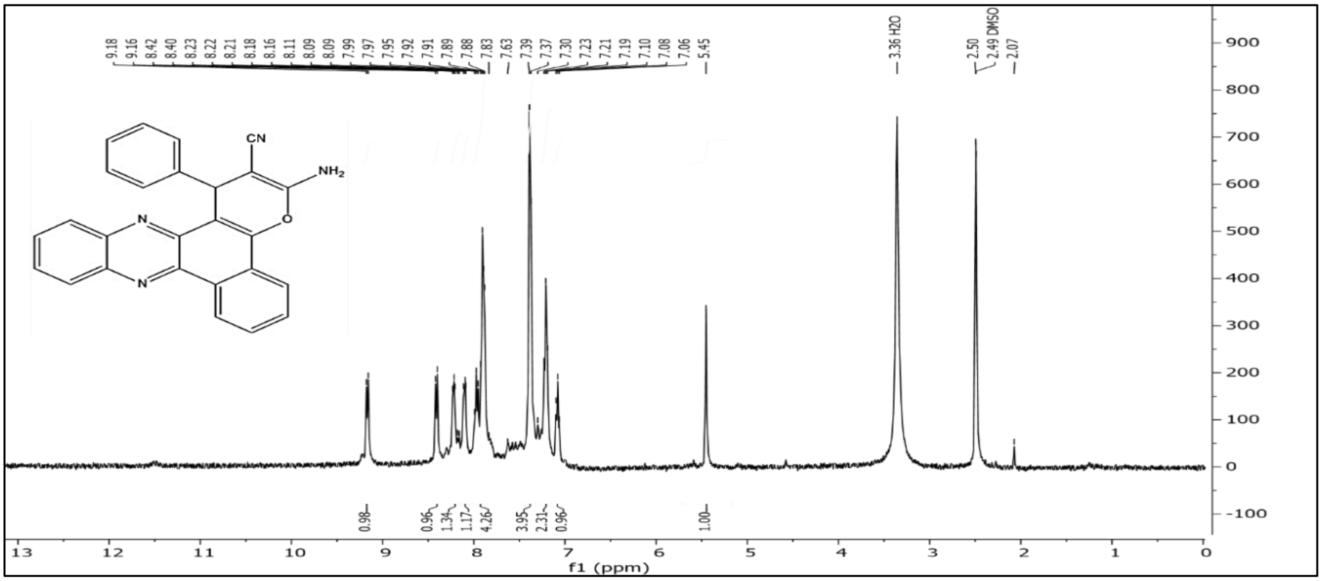

^{1}HNMR of 5a

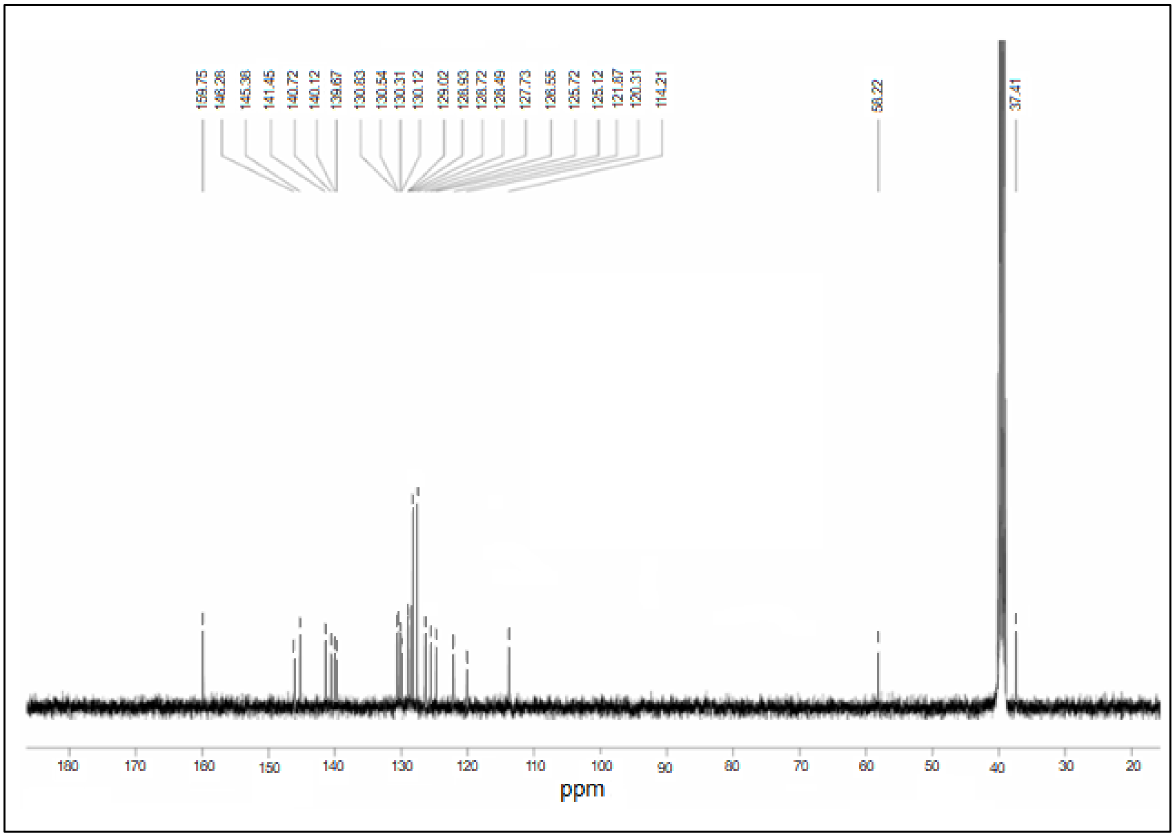

^{13}C NMR of 5a

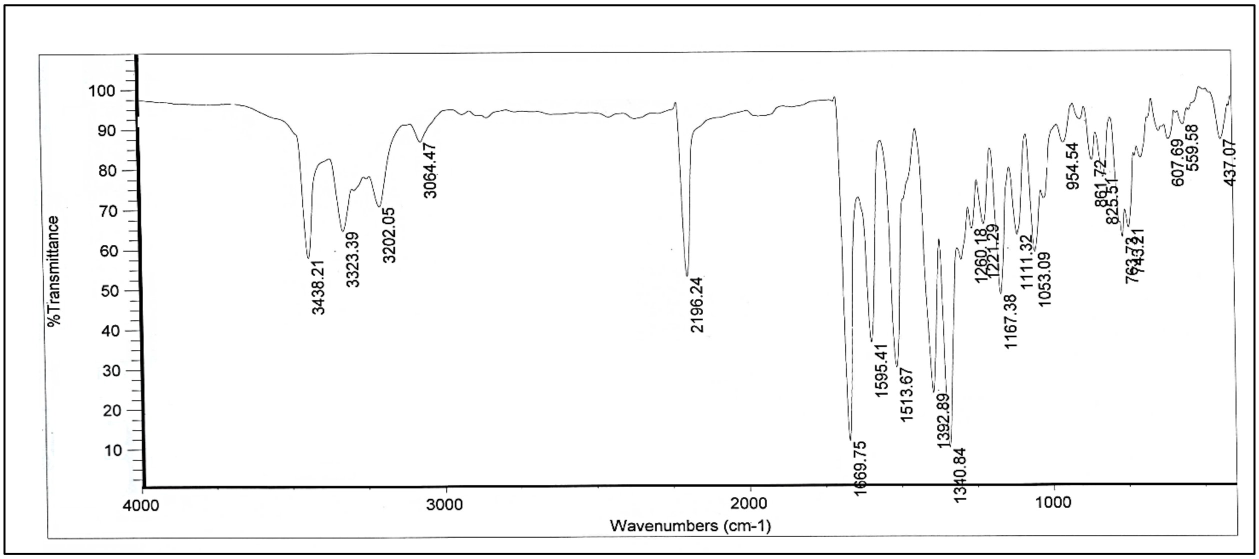

FTIR of 5b

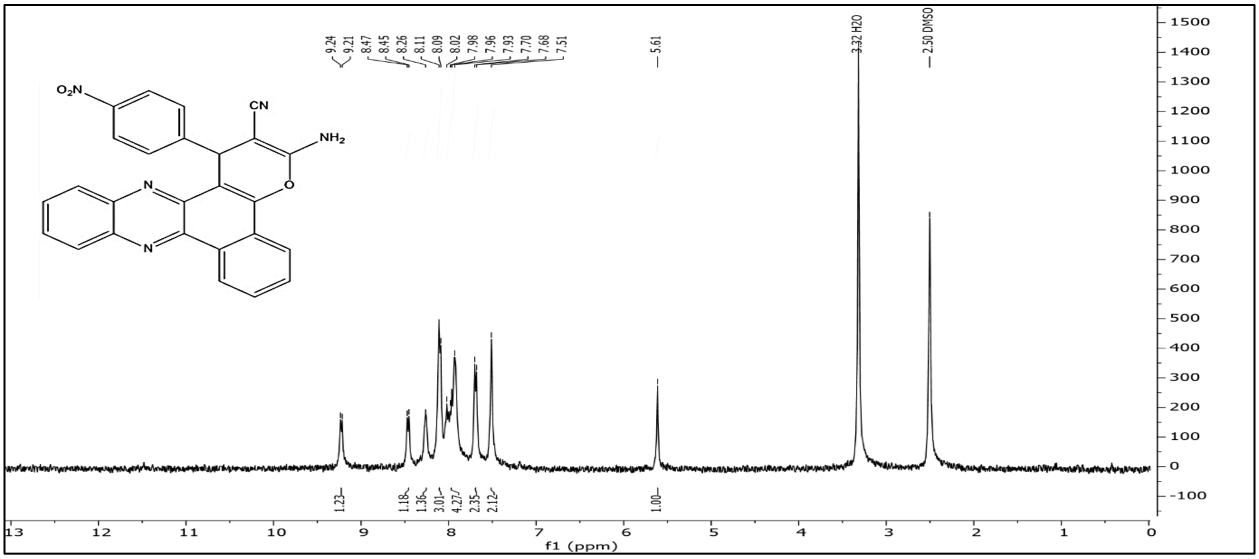

^{1}HNMR of 5b

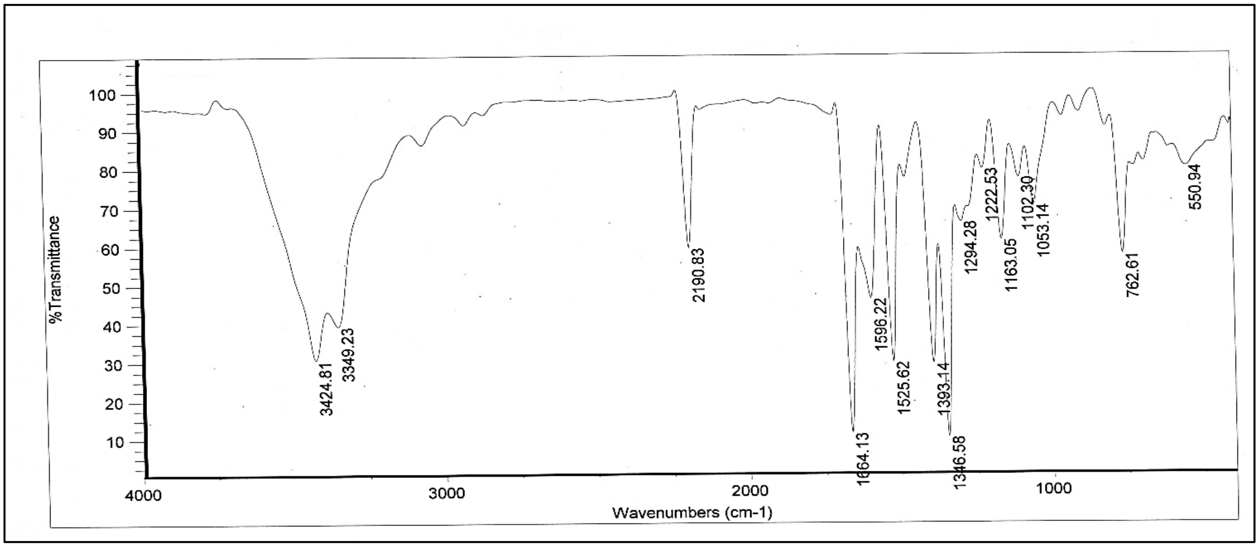

FTIR of 5c

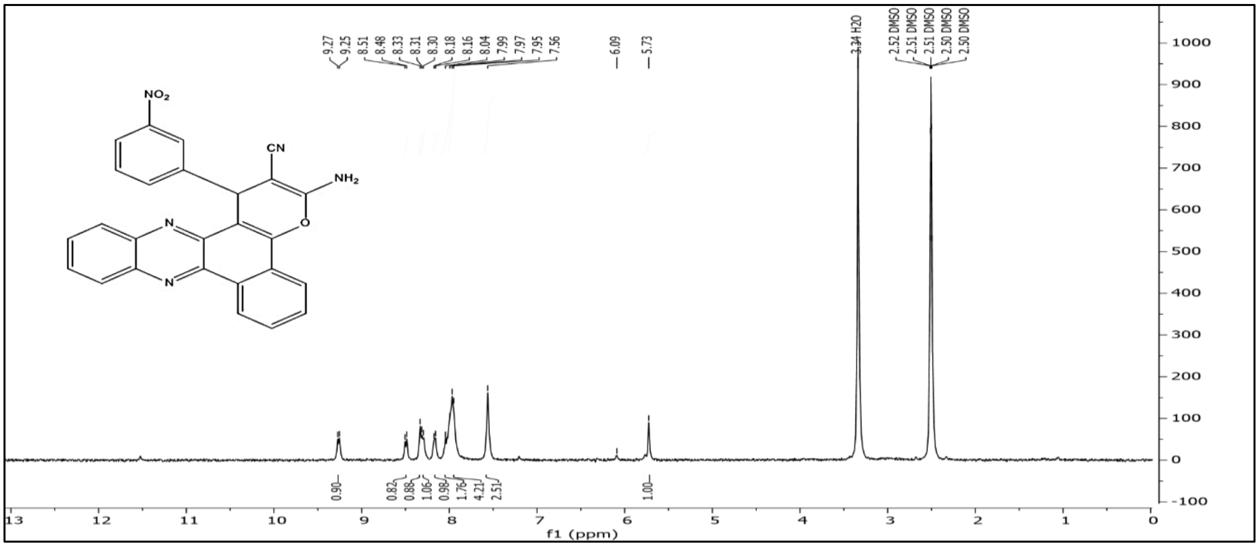

^{1}HNMR of 5c

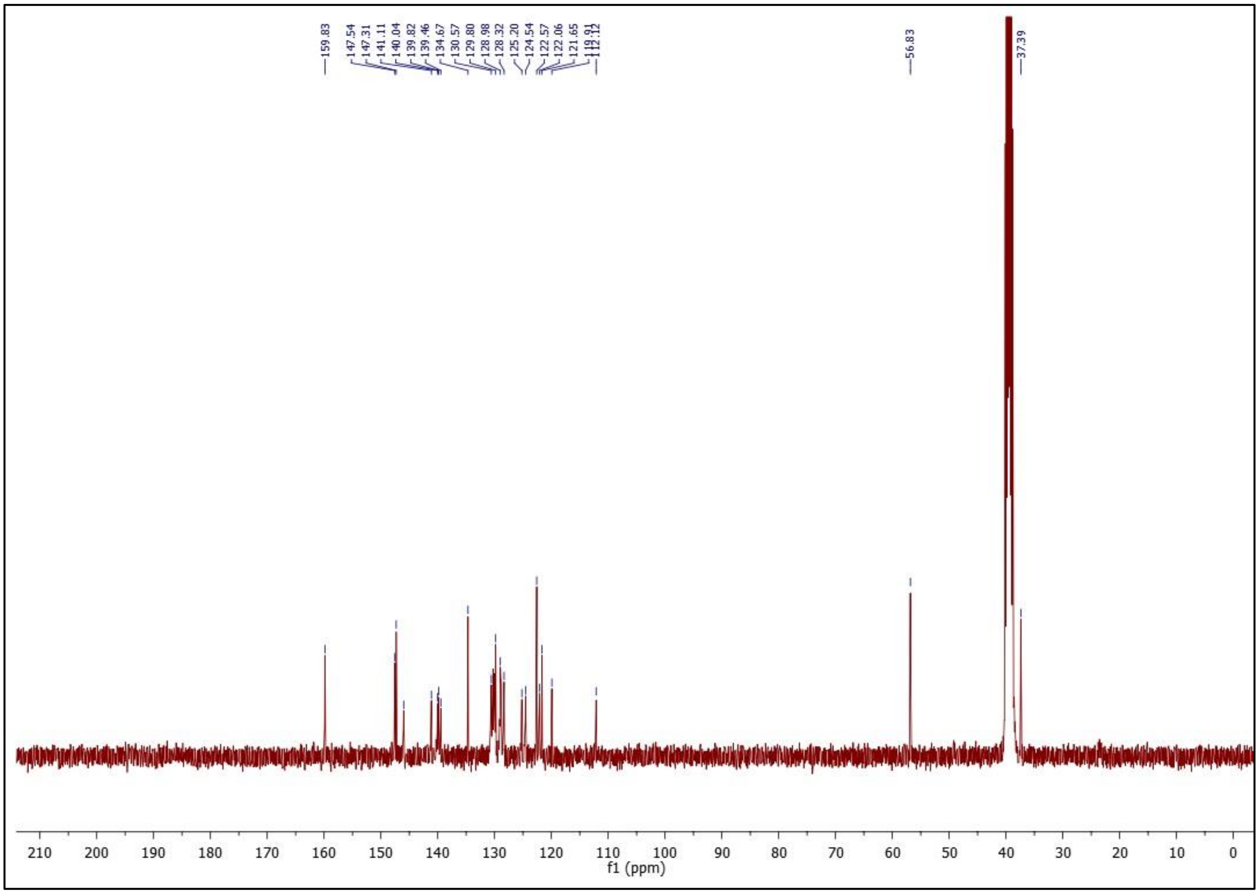

^{13}C NMR of 5c

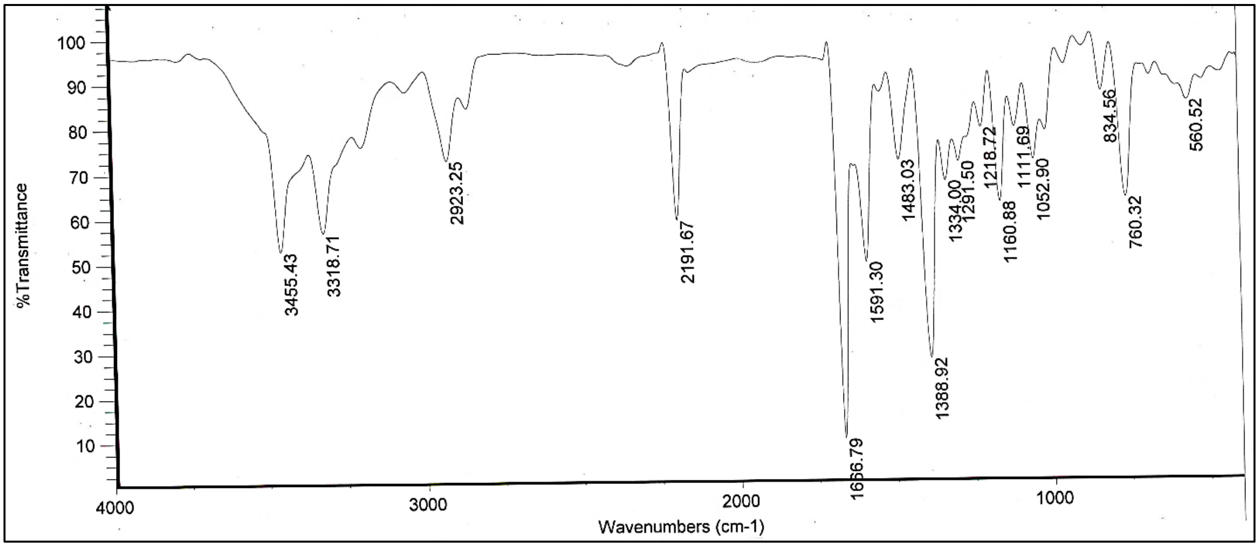

FTIR of 5d

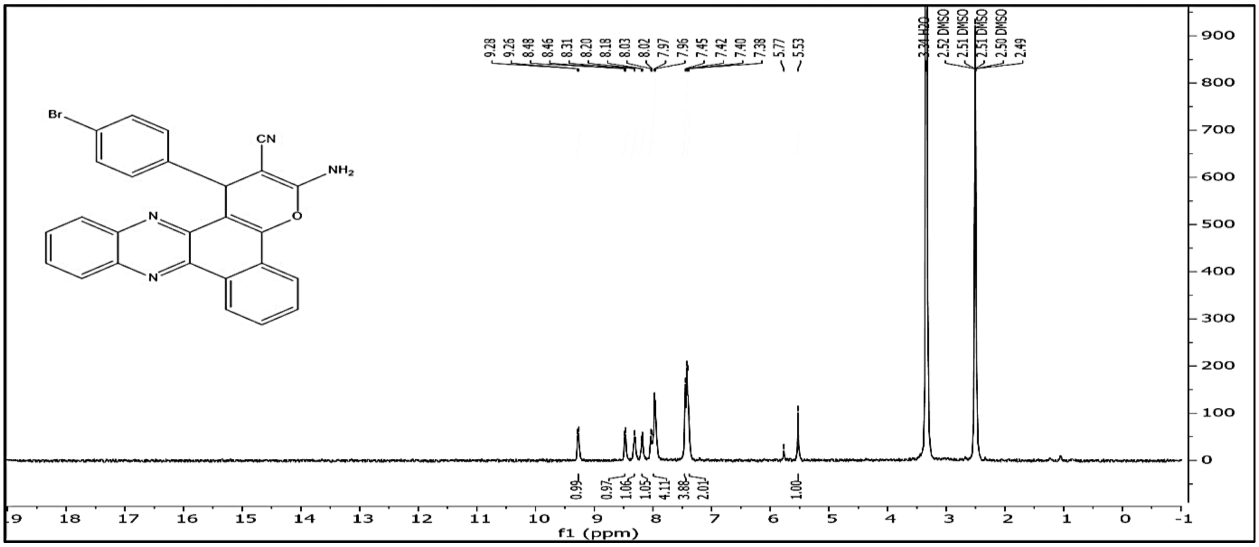

^{1}HNMR of 5d

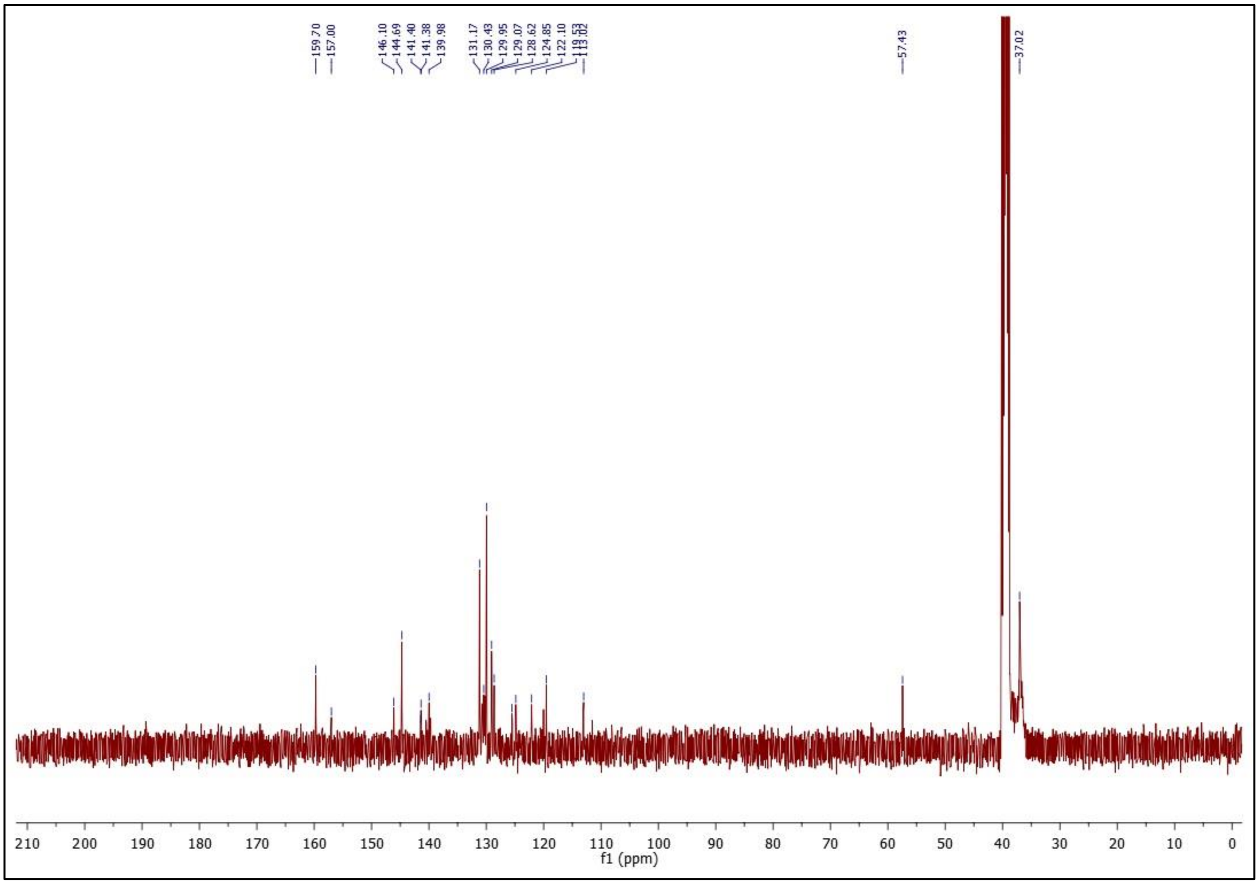

^{13}C NMR of 5d

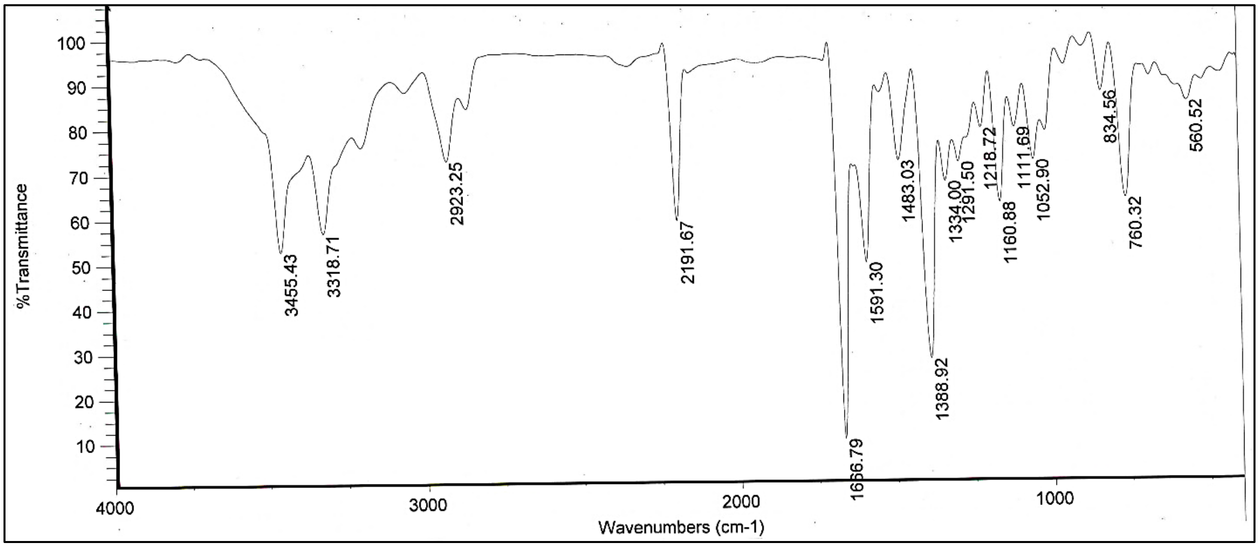

FTIR of 5e

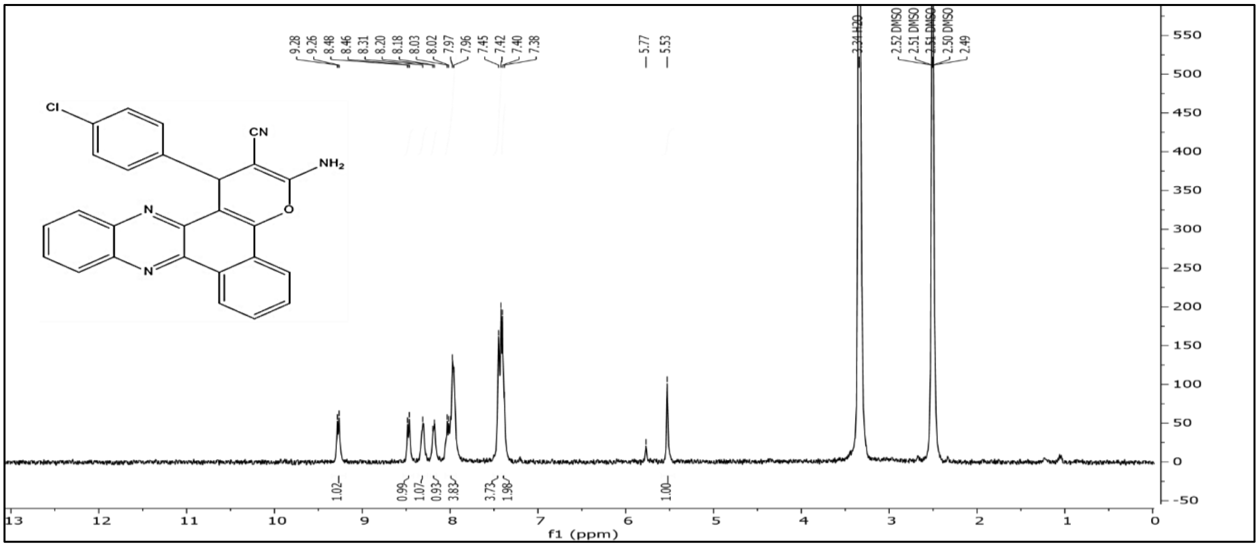

^{1}HNMR of 5e

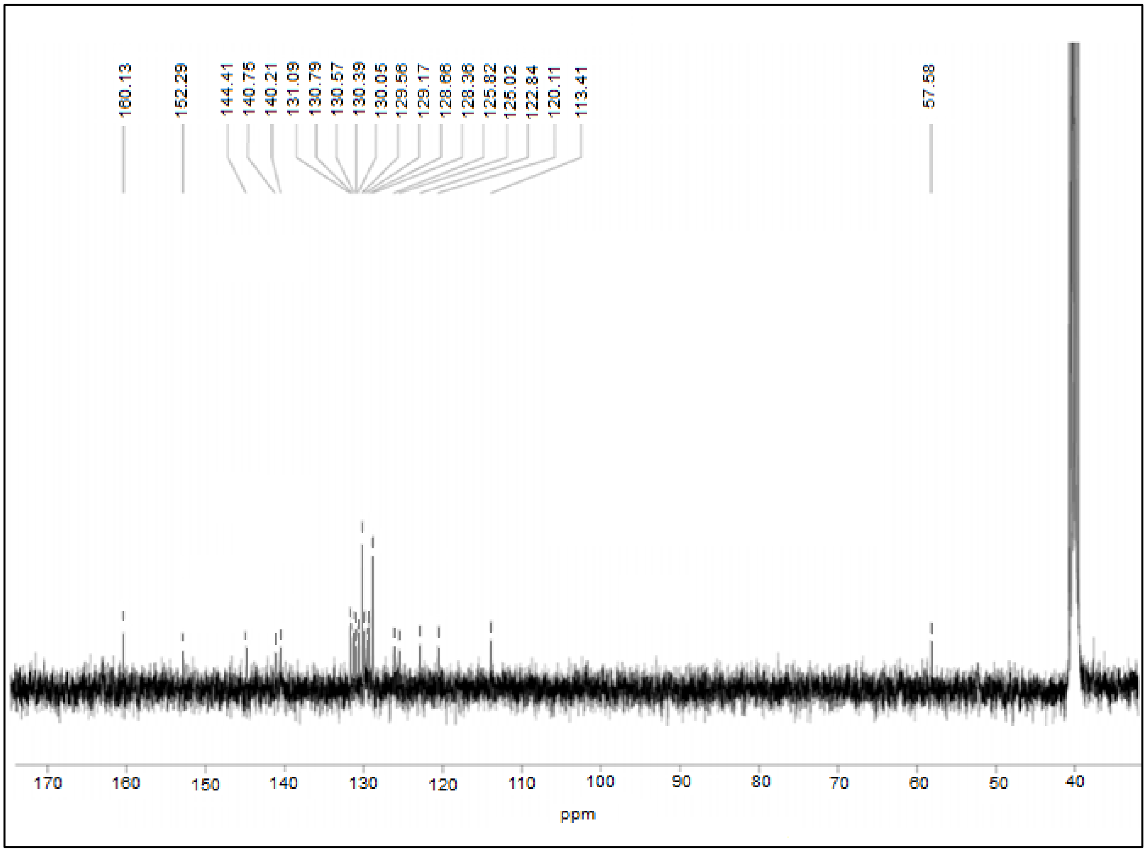

^{13}C NMR of 5e

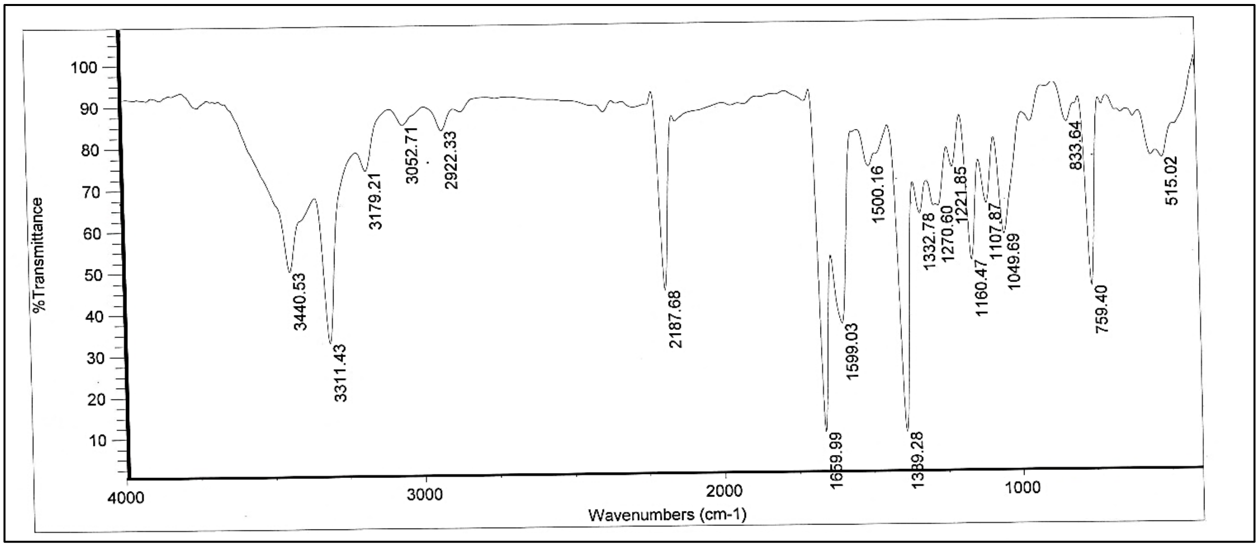

FTIR of 5f

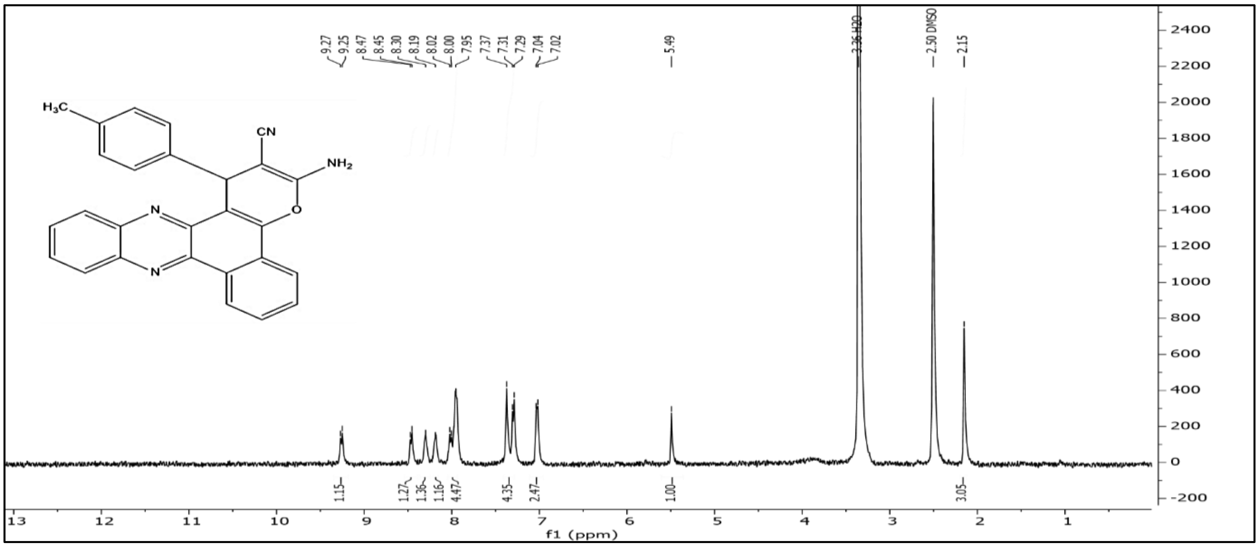

^{1}HNMR of 5f

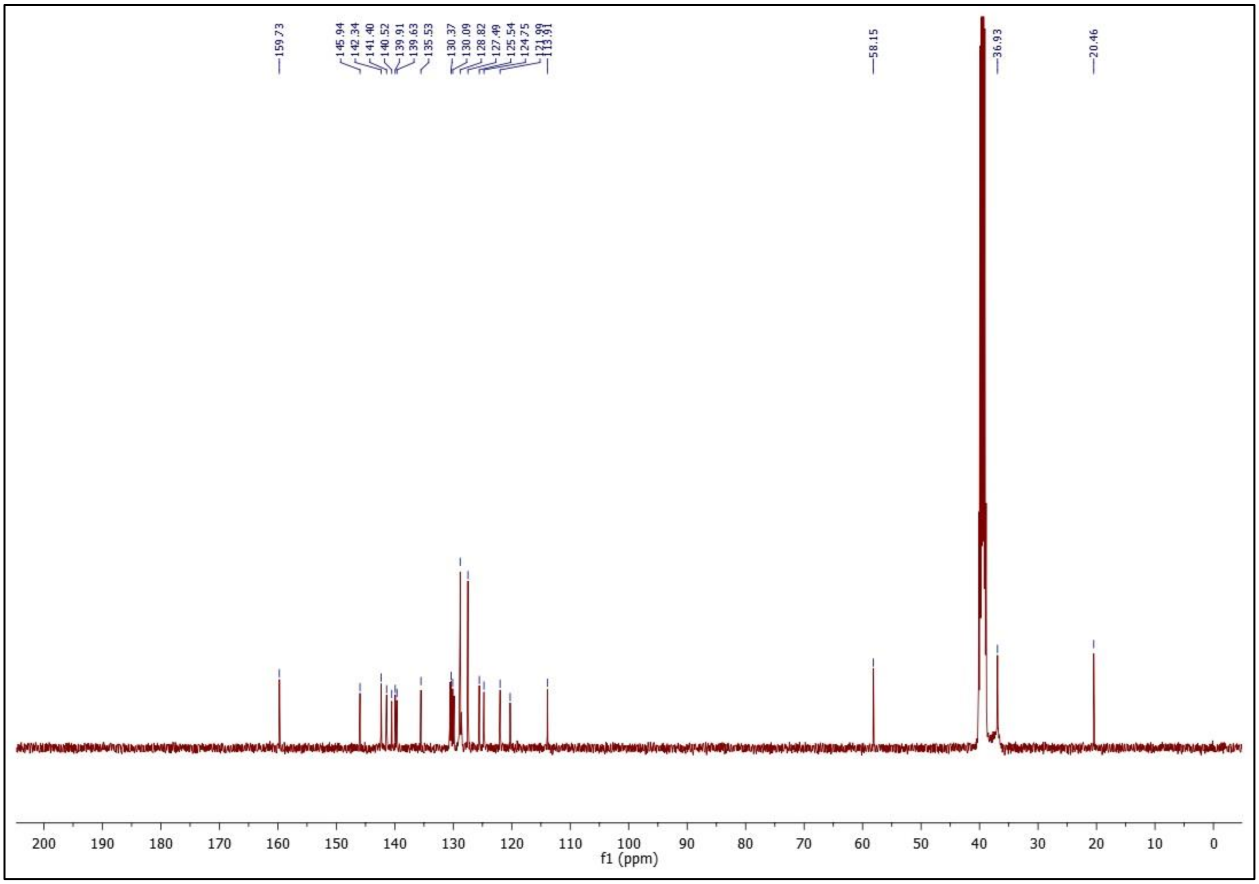

^{13}C NMR of 5f

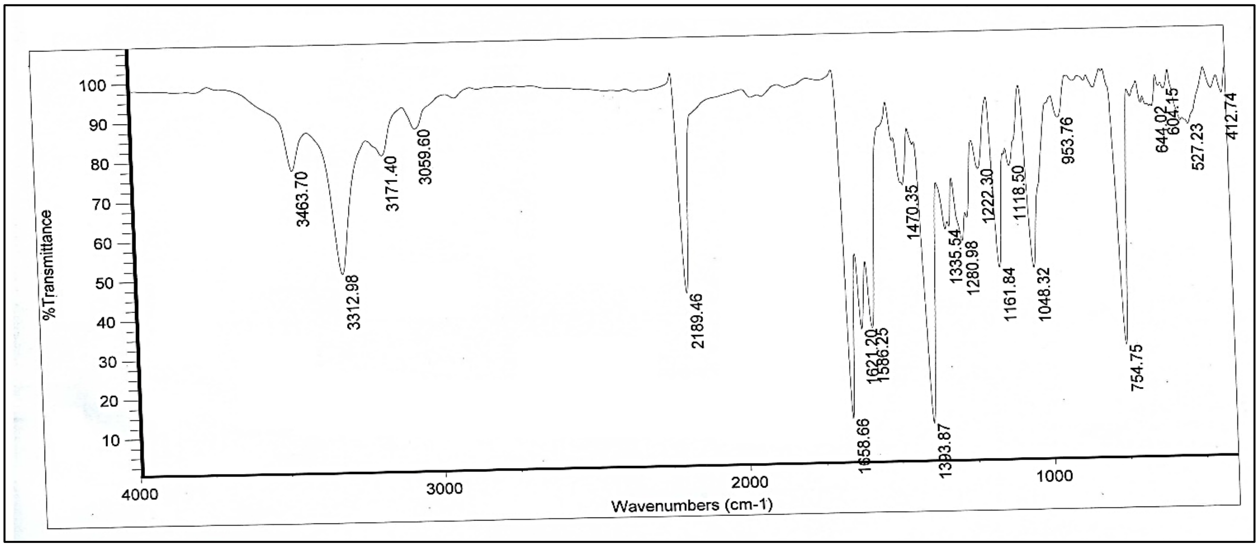

FTIR of 5g

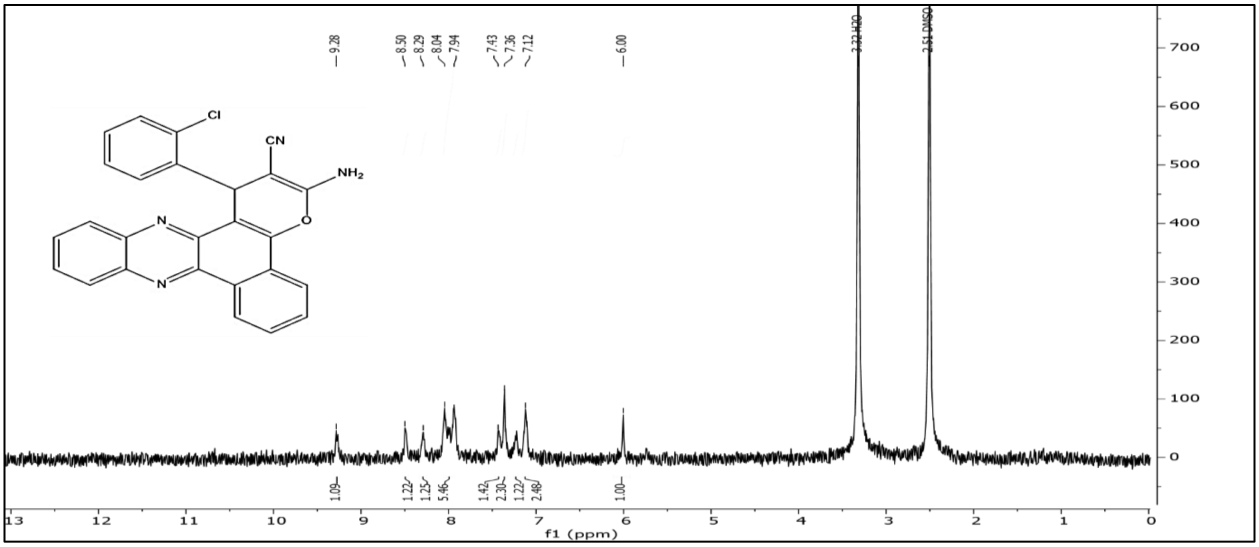

^{1}HNMR of 5g

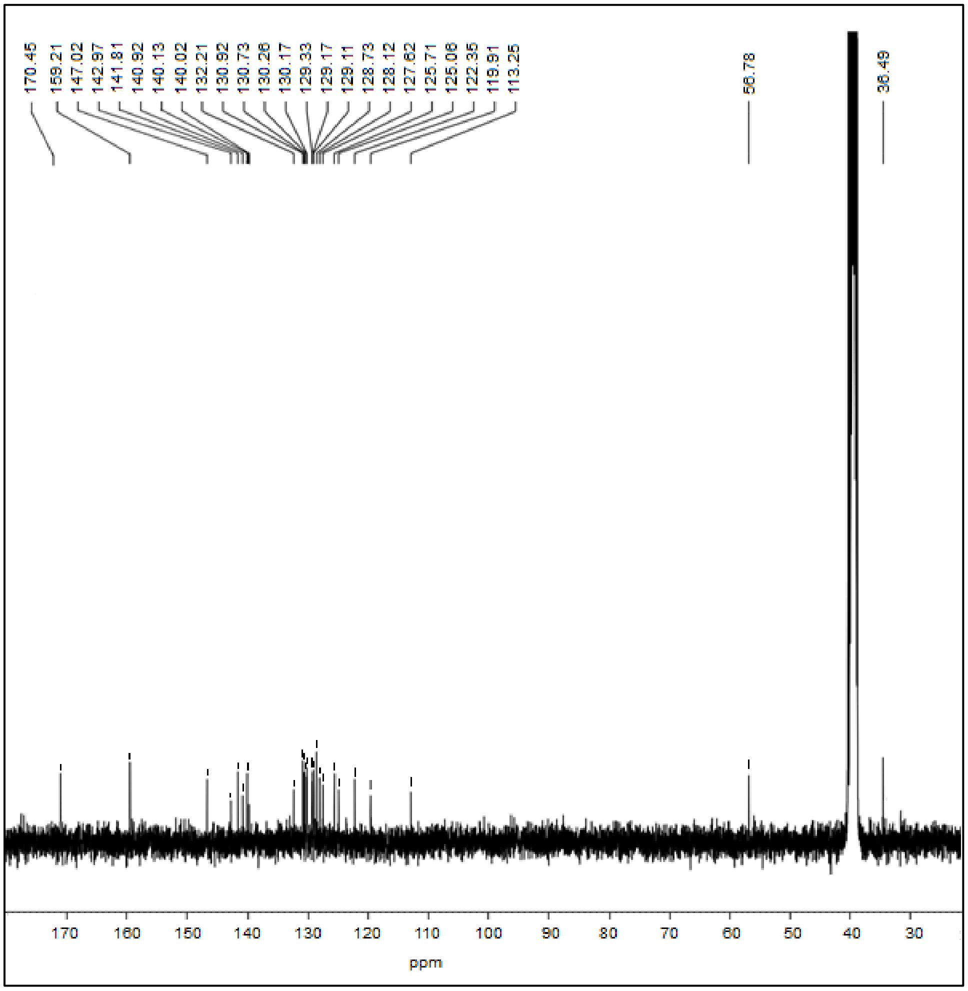

^{13}C NMR of 5g

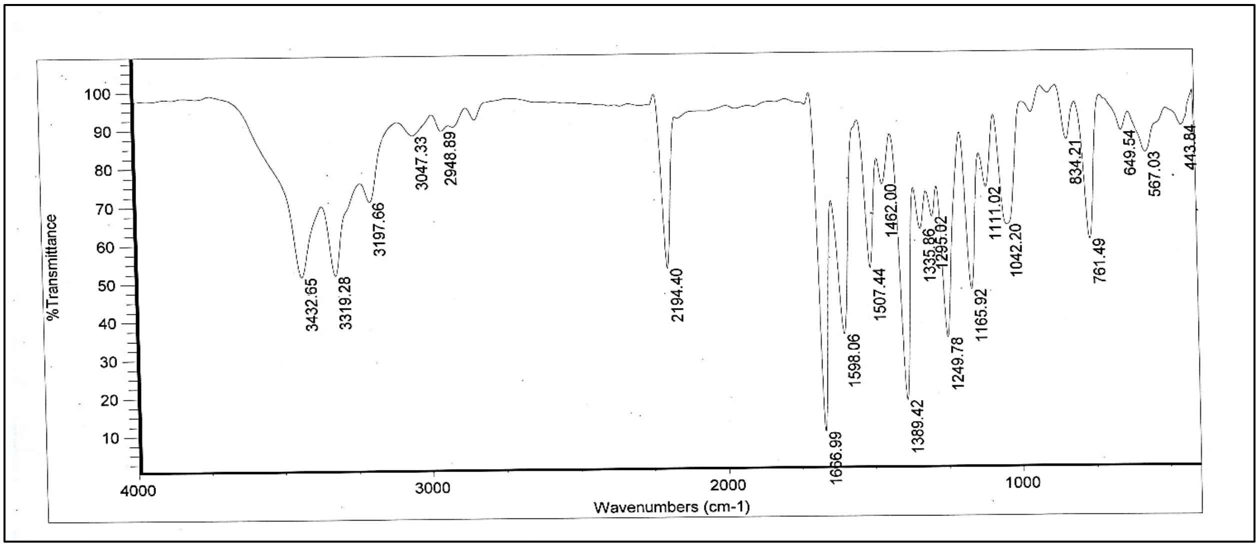

FTIR of 5h

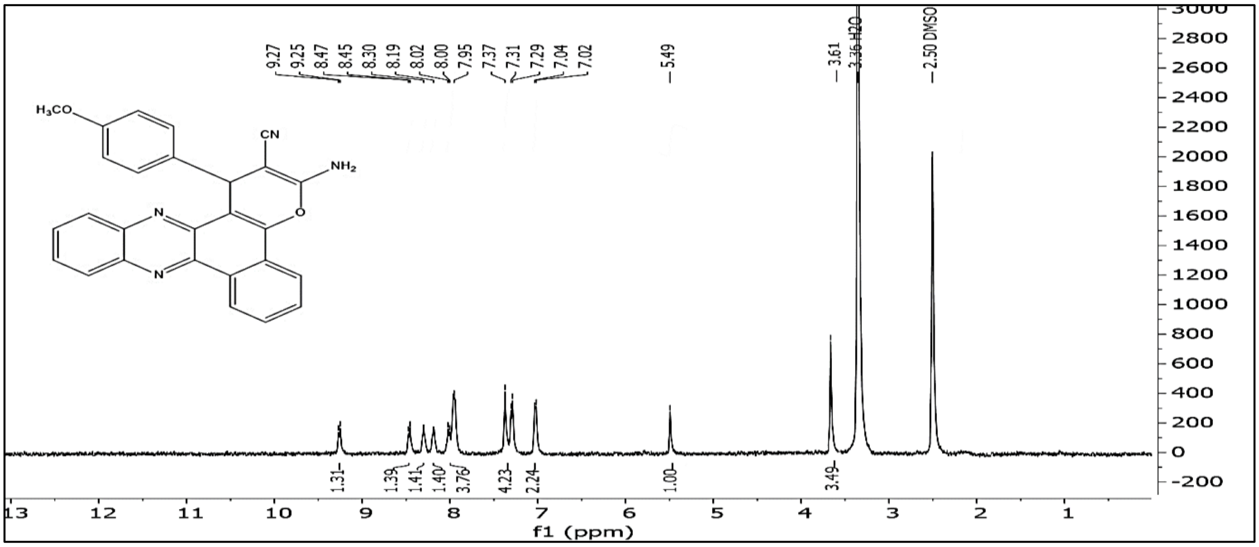

^{1}HNMR of 5h

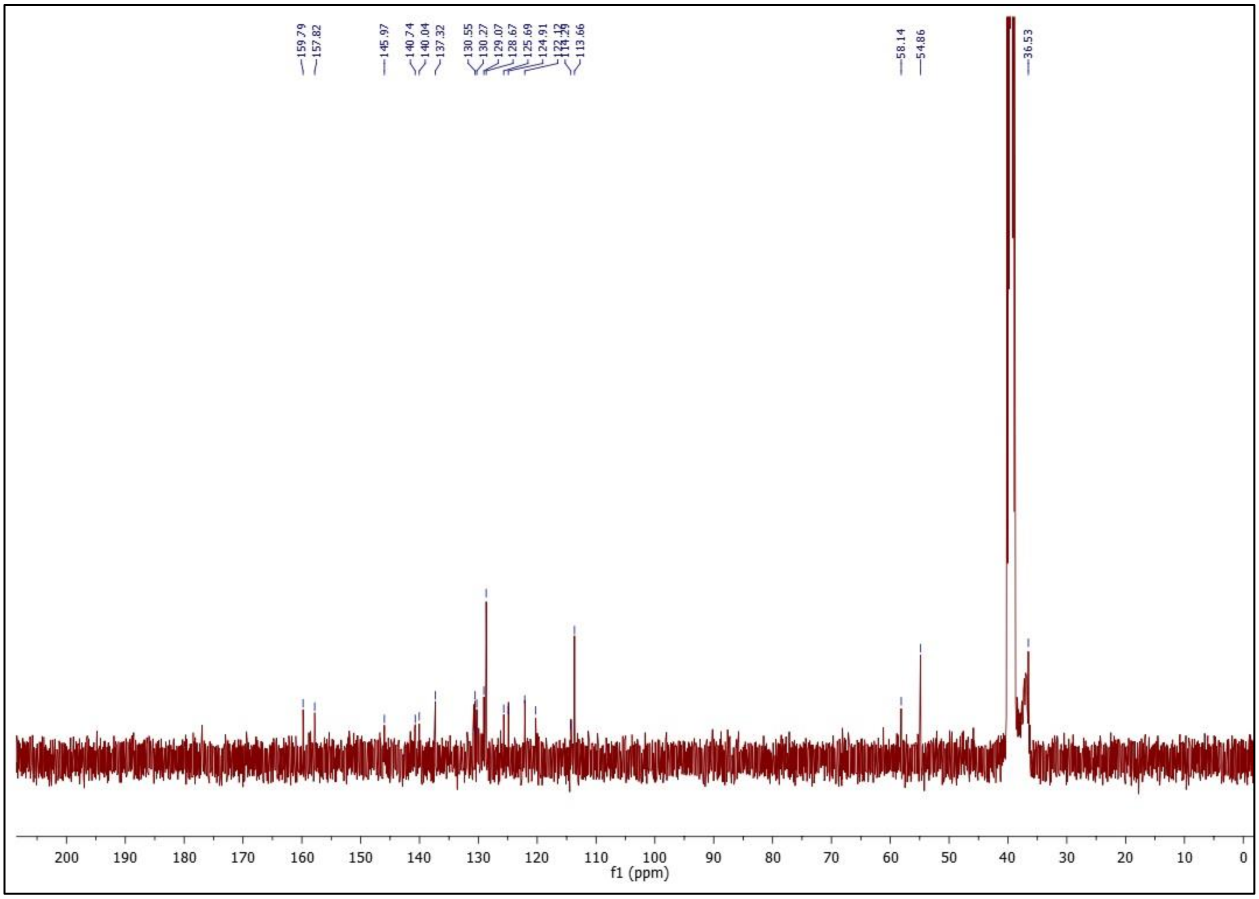

^{13}C NMR of 5h

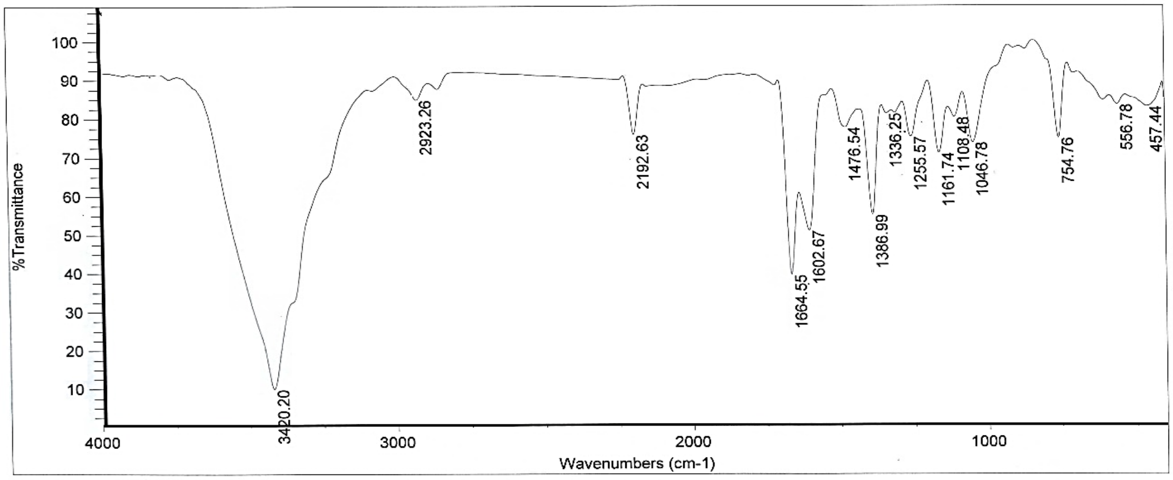

FTIR of 5i

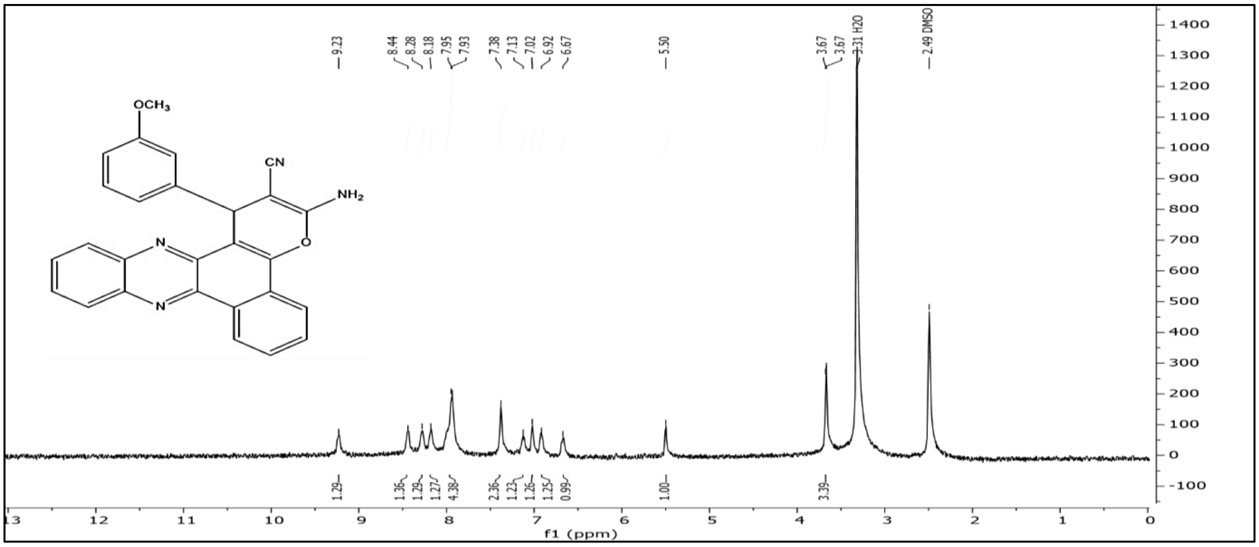

^{1}HNMR of 5i

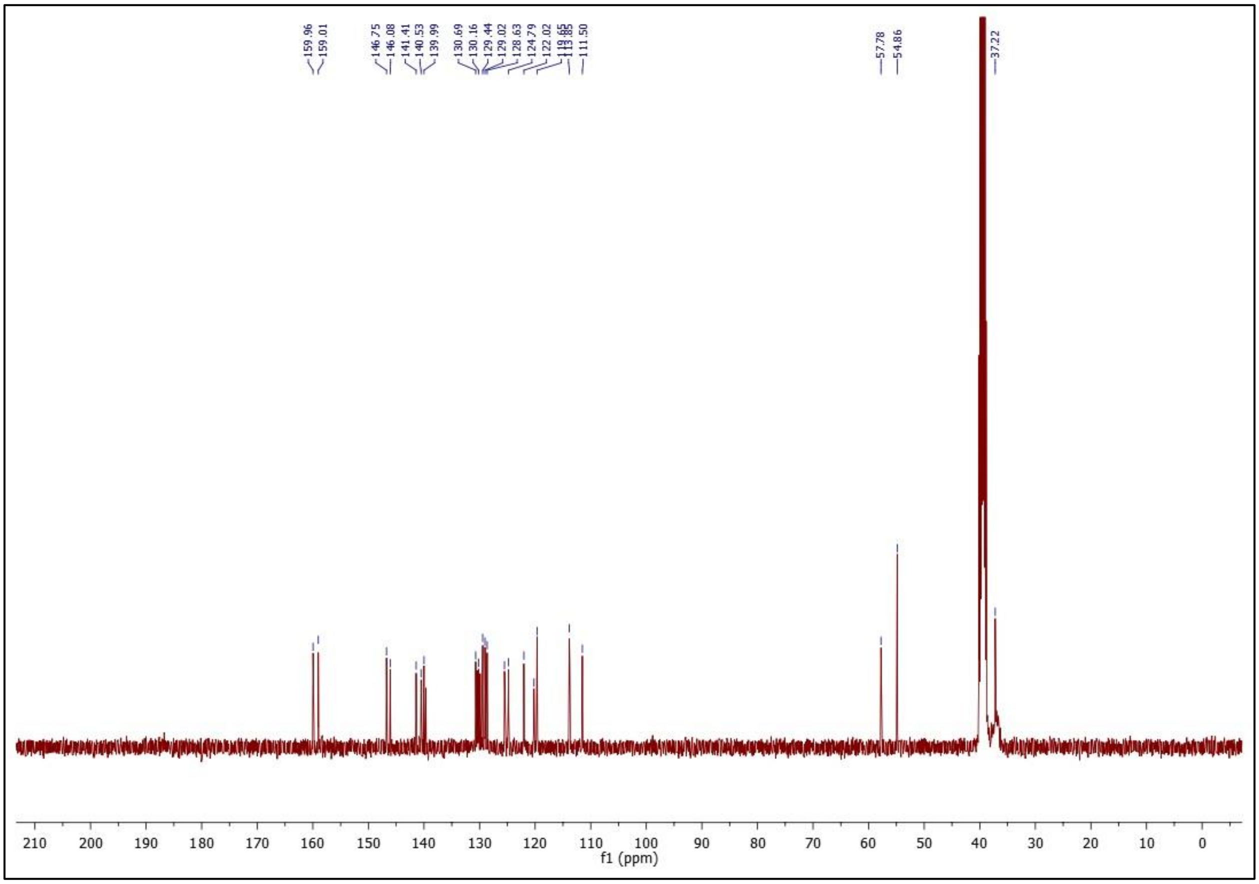

^{13}C NMR of 5i

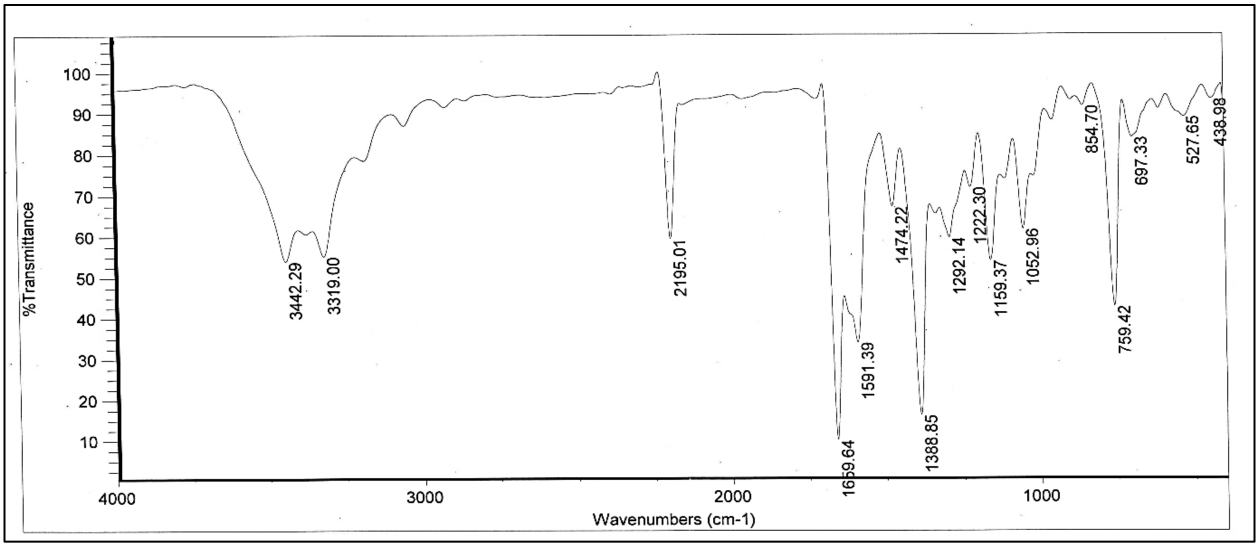

FTIR of 5j

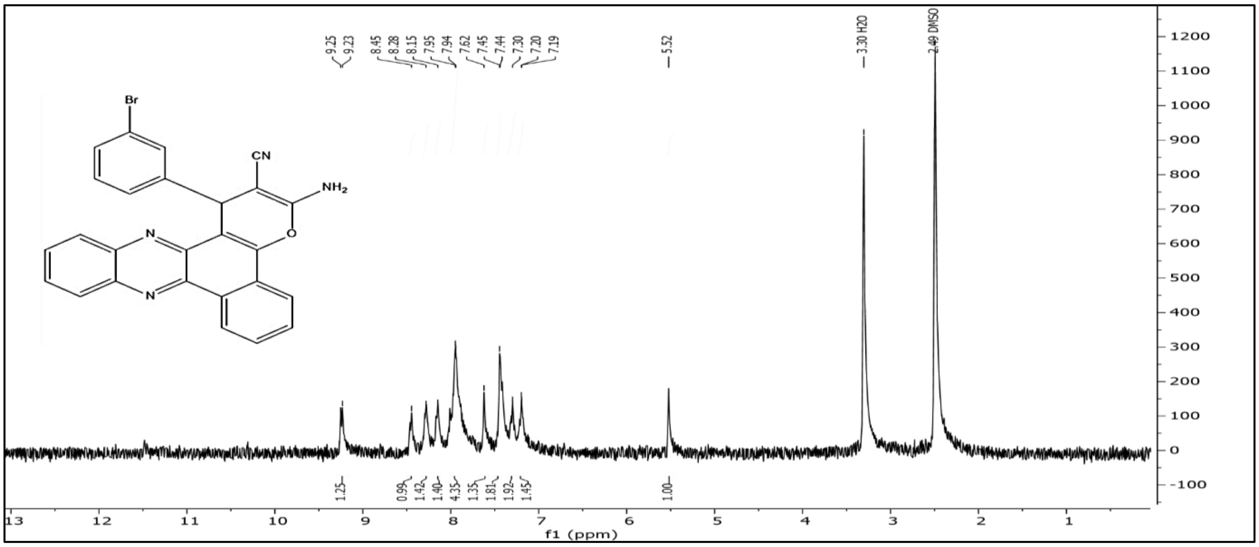

^{1}HNMR of 5j

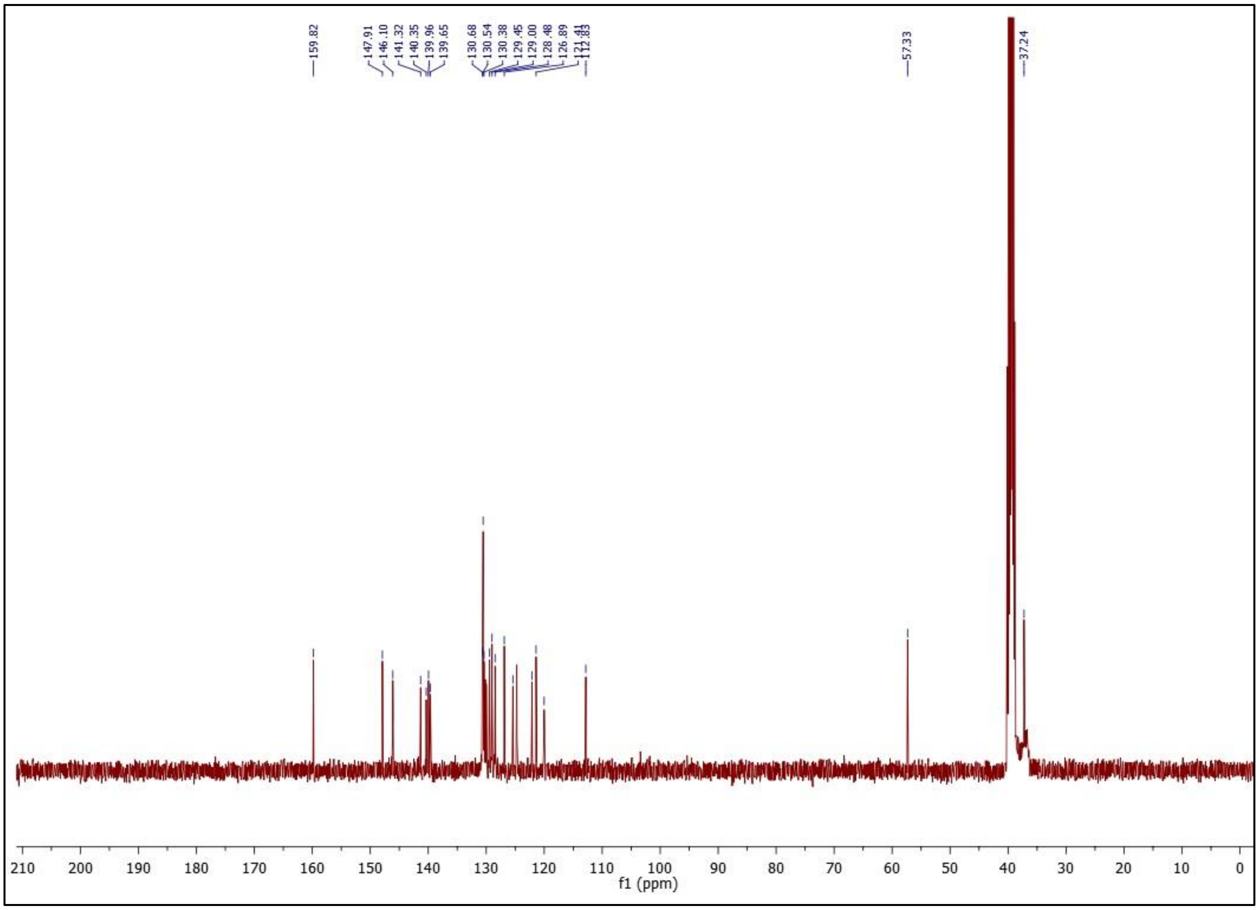

^{13}C NMR of 5j

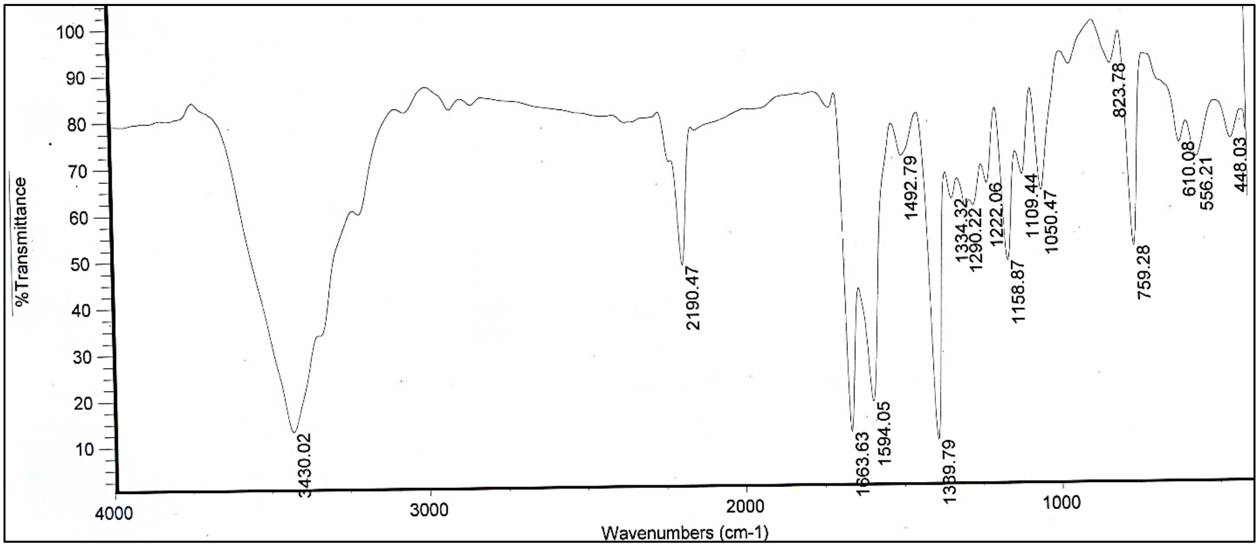

FTIR of 5k

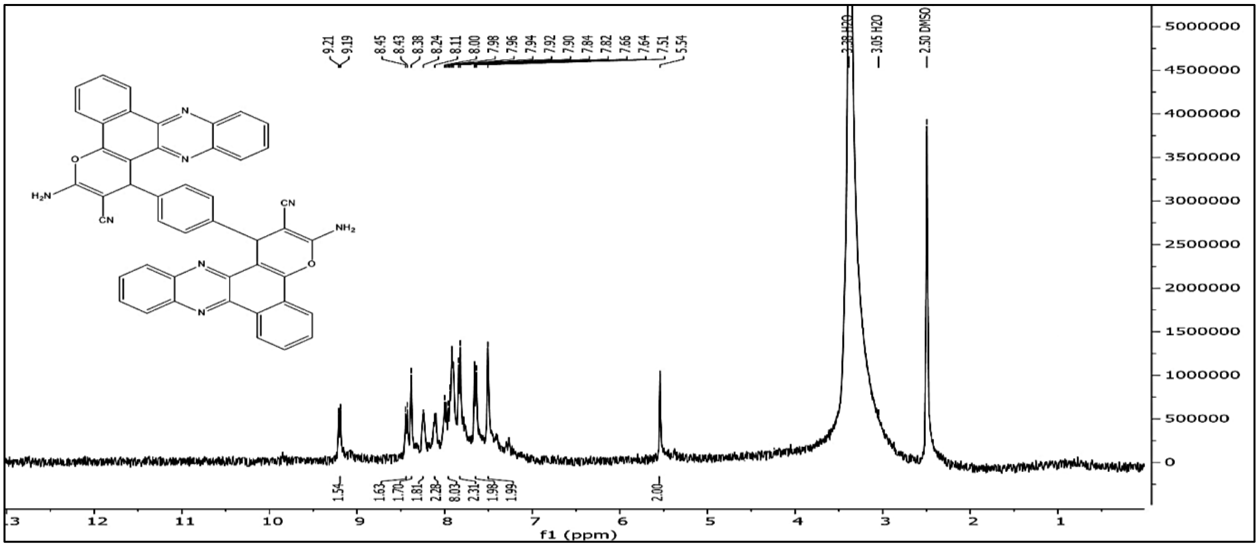

^{1}HNMR of 5k

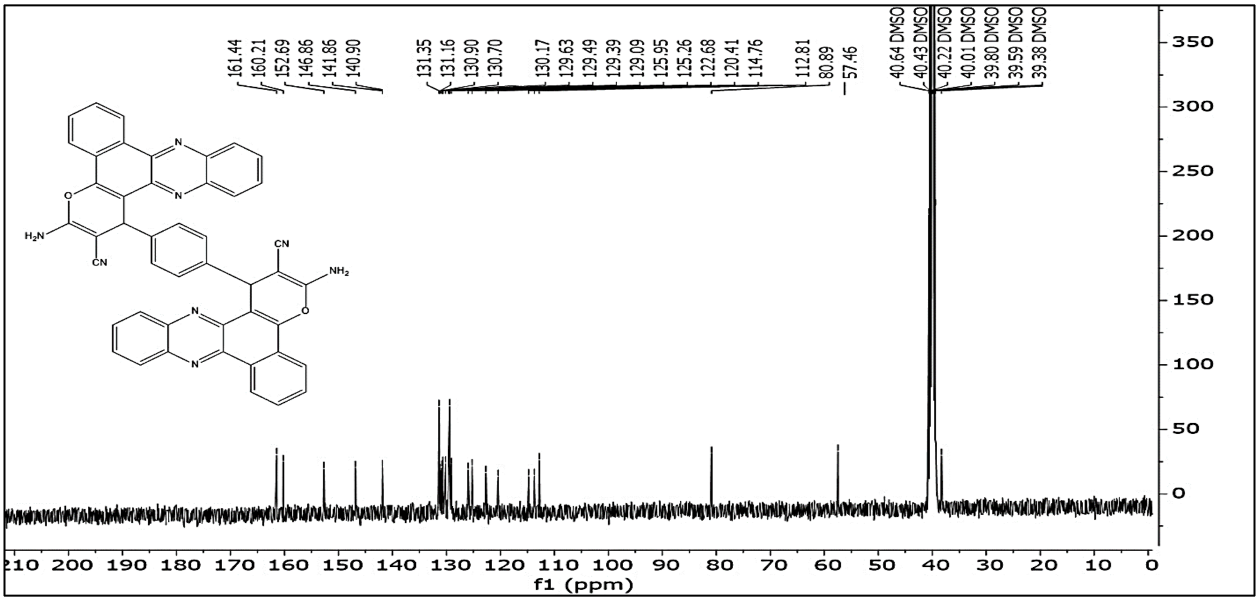

^{13}C NMR of 5k

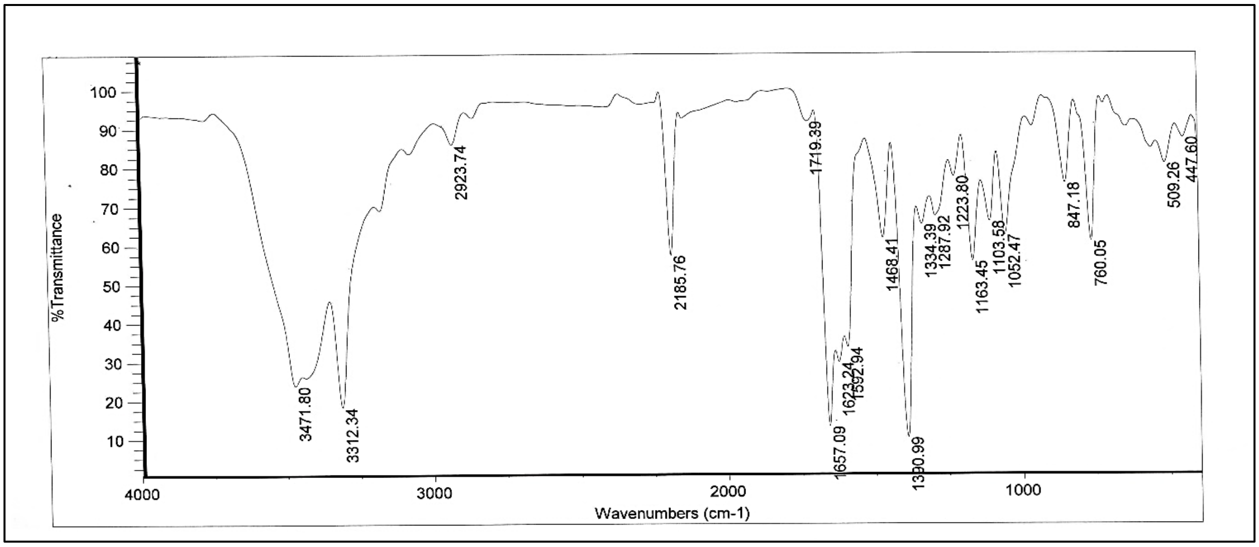

FTIR of 5l

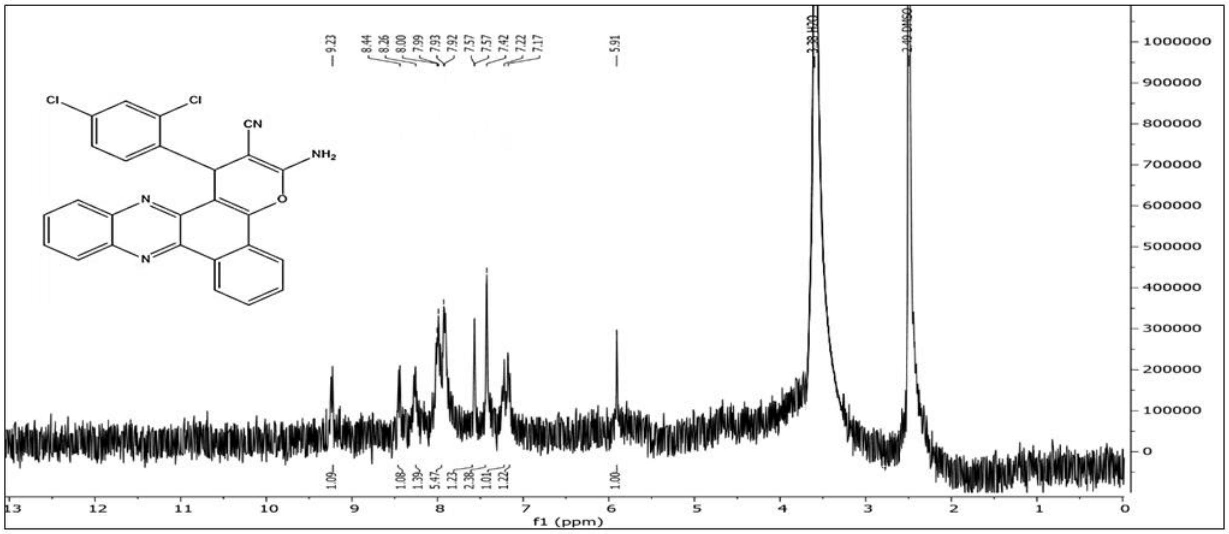

^{1}HNMR of 5l
